# Persistent loss of animal diversity on a rocky shore over nine decades and across multiple investigators

**DOI:** 10.7717/peerj.21099

**Published:** 2026-04-16

**Authors:** Robin Elahi, Fiorenza Micheli, James M. Watanabe

**Affiliations:** 1 Oceans Department, Hopkins Marine Station, Stanford University, Pacific Grove, CA, United States; 2 Stanford Center for Ocean Solutions, Stanford University, Pacific Grove, CA, United States

**Keywords:** Ecological monitoring, Biodiversity change, Historical ecology, Species richness, Rocky intertidal

## Abstract

Long-term ecological monitoring inevitably requires a ‘passing of the quadrat’ from one investigator to another. Here we present the challenge and opportunity of inferring biodiversity change over time and across investigators using a rocky intertidal case study. An intertidal transect was established and first surveyed in 1931–1933, then resurveyed in detail in 1993. After 1993, the transect was surveyed 16 more times, with surveys nested within four distinct investigator eras. We addressed two goals with our dataset. First, we used a causal framework to detect temporal change in biodiversity (species richness, Hill-Shannon and Hill-Simpson diversity, and species evenness) and then attribute biodiversity change to seawater temperature. Second, we tested the hypothesis that population change in abundance was associated with the geographic range of species. Over thirty years (1993–2023), species richness showed no linear trend but was associated with investigator era; we hypothesize that sampling effort contributed to this effect. In contrast, we observed weak linear declines in the diversity of common taxa (Hill-Shannon), dominant taxa (Hill-Simpson), and evenness over the recent thirty years, but no evidence of investigator effects. We estimated a weak negative effect of maximum sea surface temperature on Hill-Shannon diversity (−0.25 common taxa °C^−1^). Relative to the historical baseline in the 1930s, there was no evidence for change in richness but strong evidence for change in diversity metrics. On average, Hill-Shannon diversity declined by 41–61%, Hill-Simpson diversity declined by 32–56%, and species evenness declined by 41–68% since the 1930s. Declines in diversity were associated with several taxonomic and functional changes in species composition. On average, species with a southern geographic range increased in abundance more than coastwide species, reinforcing previous work on the historical transect that demonstrated a fingerprint of seawater warming. Taken together, this case study emphasizes the value of marine stations in providing a venue for sustained periodic ecological monitoring at small spatial scales, long duration, and fine taxonomic resolution.

## Introduction

Observational field studies are the foundation of ecology by providing the inspiration and context for experiments, theoretical models, empirical inference, and management. Long-term field studies are disproportionately valuable to ecology and policy (Hughes et al., 2017) and critical to the assessment of biodiversity change in a climate of anthropogenic forcing (Magurran et al., 2010). Most long-term quantitative studies are on the order of several decades (Vellend et al., 2013; Dornelas et al., 2014; Elahi et al., 2015; Kaplanis, 2023), with notable exceptions rooted in the century-long tradition of Western European natural history (Southward, Hawkins & Burrows, 1995; Richardson et al., 2006; Silvertown et al., 2006). Despite their importance, funding for long-term monitoring has declined (Hughes et al., 2017) and thus the maintenance of these studies is a challenge. Besides funding, the transfer of knowledge, methods, and data between sequential investigators is critical to extend long-term datasets beyond a few decades, the typical length of an academic career.

The potential for error associated with different investigators or observers is appreciable in studies of terrestrial plant communities (Morrison, 2016; Douda et al., 2023) and benthic marine communities (Benedetti-Cechi et al., 1996; Durden et al., 2016). The sources of observer error in ecological surveys of sessile or slow-moving assemblages typically include overlooking species, misidentifying species, and incorrectly estimating abundances of species (Durden et al., 2016; Morrison, 2016). Rare species can affect inferences about biodiversity change in long-term monitoring efforts and historical resurveys (Douda et al., 2023). In particular, the estimation of species richness is sensitive to sampling effort (Gotelli & Colwell, 2001), which can vary by observer, sampling occasion, or both. In addition to observer error, long-term monitoring studies are susceptible to spatial error (*i.e*., variability in site relocation) and seasonal error (*i.e*., variability in sampling dates or seasons) (Douda et al., 2023). Ideally, the estimation of biodiversity change over time considers all three of these broad categories of error.

After the detection and estimation of biodiversity trends, it is often desirable to attribute temporal biodiversity change to specific mechanisms to improve our understanding of natural systems and inform management strategies (Gonzalez, Chase & O’Connor, 2023; Schrodt et al., 2025). Direct pressures, or drivers, of biodiversity change include habitat modification, overexploitation, invasive species, pollution, and climate change (Pereira, Navarro & Martins, 2012). Notably, a reanalysis of a seminal meta-analysis supporting no net change in local-scale plant richness (Vellend et al., 2013) highlighted the dependence of biodiversity change on temperature and precipitation trends (Suggitt, Lister & Thomas, 2019). In a separate study, climate change and land modification were associated with increased richness, biotic homogenization, and warmer-adapted terrestrial assemblages across five decades in Britain (Montràs-Janer et al., 2024). In the ocean, temperature-driven restructuring is expected to be especially prominent due to the preponderance of ectothermic organisms and the potential for high connectivity (Sunday, Bates & Dulvy, 2012). Indeed, marine biodiversity projections based on climate trajectories and thermal tolerances support a net increase in local richness as a result of future species redistribution, particularly in temperate regions (Beaugrand et al., 2015; García Molinos et al., 2016); empirical evidence already supports local species gains in marine ecosystems (Elahi et al., 2015; Antão et al., 2020). Nevertheless, considerable uncertainty remains when extrapolating synthetic results to specific locations due to the particulars of species composition and environmental history; local knowledge remains critical for developing policy and management at a local or regional scale.

Here we addressed the challenges of detection and attribution with a case study of biodiversity change on an intertidal rocky shore. This study was initiated in 1931 and repeatedly sampled thereafter by several different lead investigators. The 90-year duration was accompanied by three key investigator-dependent challenges: species identification, variation in sampling effort, and variation in sampling season; spatial error was minimized by relocating the original quadrats with the help of maps and permanent markers. First, we applied structural causal models to detect temporal declines in biodiversity (*sensu* Magurran, 2021) and attributed those declines to increases in seawater temperature. Second, to assess a mechanism underlying the observed temperature effect, we compared the contemporary abundances of individual species to the historical baseline in the context of geographic range. That is, we re-evaluated and found support for the hypothesis that warm-adapted southern species exhibit increases in local abundance relative to coastwide species, due to warming seawater (Barry et al., 1995; Sagarin et al., 1999).

## Methods

Portions of this manuscript were published previously as part of a preprint (Elahi, Micheli & Watanabe, 2024).

### History of the intertidal survey

This study was initiated in 1931 and completed in 1933 by Willis Hewatt, a graduate student at Stanford University (Hewatt, 1934). Hewatt surveyed 108 consecutive square yards along a transect perpendicular to shore at Hopkins Marine Station (HMS), Pacific Grove, California, USA. The transect is located within the Hopkins Marine Life Refuge that has been protected as a no-take reserve since 1931, coinciding with the start of Hewatt’s research.

A primary focus of Hewatt’s work was the spatial distribution of animals along the shore; the abundances of 90 species in each quadrat were presented in his manuscript (Table 10 in Hewatt, 1937). Nineteen of these 90 species were not counted but instead categorized qualitatively as “abundant”, “common”, “occasional”, or “rare”; these qualitative designations were not associated with numerical approximations. After the surveys had been completed for the 108 quadrats, four “typical squares” were selected as representative of intertidal zones; these four quadrats were resurveyed intermittently during changing seasons. A complete list of species (*n* = 170) was also provided (Section VII in Hewatt, 1937) but the abundances of the additional species were not assessed qualitatively or quantitatively (*i.e*., they were not presented in his Table 10). Hewatt acknowledged assistance with the identification of crustaceans, gastropods (including nudibranchs), and fishes.

Bruce Provin was the first to resurvey Hewatt’s transect as part of a student project at HMS (Provin, 1949). Provin relocated (using permanent markers and maps) and resurveyed four of Hewatt’s “typical squares” (quadrats 12, 24, 35, 90). All macroscopic organisms were counted and presented in the manuscript; those that couldn’t be counted exactly were rated as “abundant”, “common”, “occasional”, or “rare”; some of these species overlapped with Hewatt’s qualitative designation, but not all. Species were identified using *Between Pacific Tides* (Ricketts & Calvin, 1948), *A Laboratory and Field Textbook of Invertebrate Zoology* (Light, 1941), and various monographs and articles; help was also obtained from the course instructors and other students.

Between 1993 and 1996, Hewatt’s transect was again resurveyed by undergraduate students Rafe Sagarin and Sara Gilman, and their mentors, Charles Baxter and Jim Barry; hereafter referred to as SBGB (Sagarin et al., 1999). Sagarin and Gilman relocated and resurveyed 57 plots between spring 1993 and summer 1995. During summer 1996, SBGB resurveyed the first 19 plots surveyed in spring 1993; this included quadrats 27–38 and 62–68 (these 19 quadrats are hereafter referred to as ‘core’ quadrats). With few exceptions, all individuals within a plot were counted, including those on or under marine macrophytes or other species. Species that could not be readily and nondestructively identified were not counted; only species that could be identified with the unaided eye were counted (Sagarin et al., 1999). Regional guides were used for species identification (Smith & Carlton, 1975; Morris, Abbott & Haderlie, 1980).

To continue the long-term monitoring effort between 1999 and 2015, Sagarin returned to HMS intermittently to resurvey the core quadrats. These resurveys occurred in 1999, 2002, 2005, 2009, 2014, 2015. No description of methods is available; we assume that the methods were the same as the methods described by SBGB for the years 1993 and 1996. Field notes indicated that within a given year, quadrats were occasionally sampled in different seasons.

In 2015, Sagarin died in a tragic vehicle collision while cycling. Fiorenza Micheli and James Watanabe began surveying the core quadrats in 2016 to maintain Sagarin’s efforts; we now refer to the study as the Hewatt-Sagarin transect to honor Sagarin’s memory and his dedicated support of observational ecology (Sagarin & Pauchard, 2010). Surveys were conducted annually between May and July and the sampling effort often included students from an undergraduate spring course at HMS. The sampling methods described by SBGB (Sagarin et al., 1999) were maintained. Regional guides were used for species identification (Morris, Abbott & Haderlie, 1980; Carlton, 2007), with assistance from local experts (*e.g*., Chuck Baxter, Jim Carlton, John Pearse).

Robin Elahi began leading the survey effort in 2020, with transfer of knowledge from Micheli and Watanabe in 2019. In addition to the core quadrats, Elahi began surveying quadrats 12, 16, 20, and 24 (hereafter referred to as ‘extra’ quadrats) to address biodiversity change at higher tidal heights in the context of climate change and sea level rise. In addition to the annual late spring sampling, a subset of quadrats was also sampled during winter months. The sampling methods remained the same, with a few exceptions (detailed in Appendix S1). Regional guides were used for species identification (Morris, Abbott & Haderlie, 1980; Carlton, 2007).

**Figure 1:**
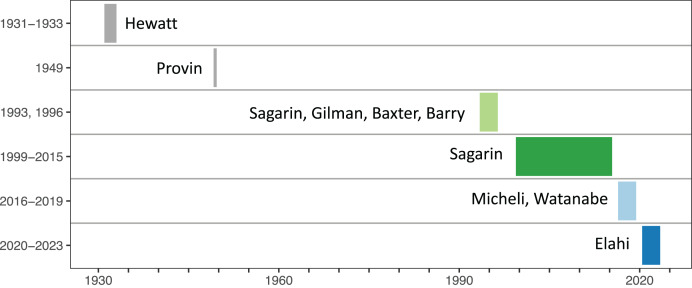
Timeline of sampling on the Hewatt-Sagarin transect, categorized by lead investigator. The width of bars spans the first and last year of sampling by each investigator and does not imply that sampling occurred in every year. The historical survey was initiated in 1931–1933 by Willis Hewatt. A small subset of permanent quadrats (*n* = 4) was resurveyed in 1949 by Bruce Provin, but these data were not considered in this manuscript. Instead, we focused on the recent three decades (1993–2023), during which 19 permanent quadrats on the historical transect have been resurveyed 17 times.

We divided the survey efforts into six investigator eras (Fig. 1), because we hypothesized that differences in species identification and sampling effort could affect inferences of biodiversity change over the ninety years of sampling. For this study, we included years from five investigator eras when all nineteen core quadrats were sampled, omitting the 1949 era because Provin sampled only three of these core quadrats. Additional details on sampling methods are provided in supplementary information (Appendix S1), including a visualization of the relative positions, tidal heights, and vertical relief of quadrats along the Hewatt-Sagarin transect (Fig. S1).

### Harmonizing taxonomy and processing the raw data for quantitative analysis

The qualitative and quantitative observations across all investigator eras were assembled into a database. For species listed in our database, we tabulated the scientific names used by Hewatt (1937) and Sagarin et al. (1999), reconciling them with current names provided in the World Register of Marine Species (WoRMS Editorial Board, 2023) (Table S1). For example, the whelk *Acanthinucella punctulata* (J.E. Gray, 1835) was recorded as *Acanthina punctulata* (J.E. Gray, 1835) and *Acanthina lapilloides* (Conrad, 1837) by Sagarin et al. (1999) and Hewatt (1937), respectively. Prior to harmonizing the taxonomy, the raw database contained 238 taxa, whose abundances were assessed either qualitatively or quantitatively at least once over the entire duration of the study.

Once we synonymized the species names in the database, we filtered the data for quantitative analysis. First, we removed taxa that were not counted, but whose abundance was scored qualitatively by Hewatt. This included amphipods (*Ampithoe* spp., *Atylopsis* spp., *Melita palmata*), spirorbid tubeworms (*e.g*., *Spirorbis* spp.), polychaetes (*Nereis* sp., *Lumbrinereis* sp.), and hermit crabs (*Pagurus* spp.). Limpet epibionts (*Lottia asmi*, *Lottia* spp.) on trochid snails (*Tegula* spp.) were also removed because they were not assessed by Hewatt. We also omitted animals recorded at taxonomic levels above genus, which started in 2020. Finally, we removed animals that were recorded as genera when more than one species was recorded within that genus, to avoid overestimating diversity; this applies primarily to 2020 and after, when genera and higher clades began to be counted. In cases where a species (or genus) was recorded in 1993 or after, but the abundance was not quantified by Hewatt, we checked to see whether Hewatt had recorded the species in the complete list (Appendix X in Hewatt (1934)). If it was not on the list, we recorded it as absent from Hewatt’s study (*i.e*., we treated it as a zero); if it was on the list, then we recorded it as present in Hewatt’s study. The final consideration for quantitative analysis was the potential for ambiguities in species identification across investigator eras. For each taxon listed in Table S1, we identified the taxonomic level at which we felt comfortable inferring patterns of biodiversity change and summarized the abundances to that level.

### Inferring biodiversity change: a causal framework

Our inferential goals in this study included both detection and attribution of biodiversity change (Gonzalez, Chase & O’Connor, 2023; Schrodt et al., 2025). One approach for attribution with observational data is the framework of causal inference (Pearl, 2010). Causal inference is commonly practiced in disciplines where experimentation is difficult or unethical (*e.g*., economics, public health), and has recently garnered attention in ecology (Arif & MacNeil, 2022; Byrnes & Dee, 2025; Schrodt et al., 2025; Siegel & Dee, 2025). This framework begins with an explicit specification of the causal pathways between variables of interest, and uses graphical rules to build appropriate statistical models for the estimation of causal effects (Pearl, 2010). Importantly, the application of structural causal models reduces bias in the estimation of ecological effects and can fundamentally alter the interpretation of ecological studies (Rinella, Strong & Vermeire, 2020; Dee et al., 2023).

**Figure 2:**
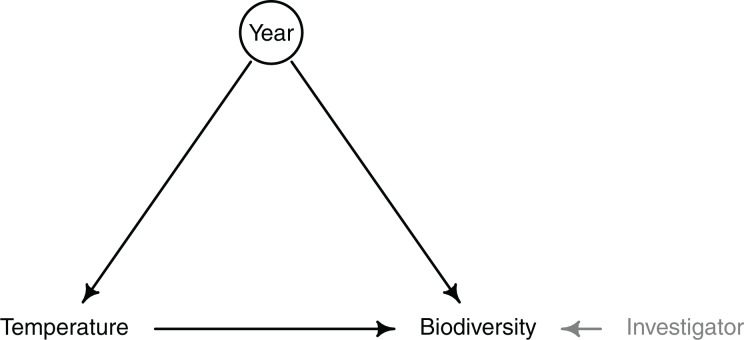
Directed acyclic graph (DAG) representing the hypothesized causal relationships between year, seawater temperature, and a metric of biodiversity. The influence of investigator on biodiversity is in grey because is not a causal path, but rather influences the estimation of biodiversity through observer effects. Year is enclosed in a circle because it represents potential unmeasured confounds that covary with year, apart from the effect of interest. In this study, we estimated the total effect of year on several biodiversity metrics, achieved by ignoring temperature (and any other environmental factor covarying with year) in our statistical model. Then, we estimated the effect of temperature on one biodiversity response by including the effect of year in the statistical model, thereby accounting for unobserved confounds.

In this study, we first wanted to detect biodiversity change over time while acknowledging the potential effect of investigators; second, we wished to attribute biodiversity change to sea surface temperature, a hypothesized driver. We represented our hypotheses with a directed acyclic graph (DAG; Fig. 2). In this DAG, we assume that time (year) can be related to biodiversity change through stochastic fluctuations of individuals, *i.e*., ecological drift (Vellend, 2016). We also assume that year causes biodiversity change through covarying mechanistic influences, which can also be thought of as temporally variable selection (Vellend, 2016). In our case, we illustrate this principle using sea surface temperature. Finally, we assume that investigators will affect the estimation of biodiversity through observer effects, which we do not consider to be causal. Our hypothesized DAG leads logically to two distinct causal models (McElreath, 2020). First, to estimate the total effect of year on biodiversity, temperature (or any other predictor that covaries with year) should not be included in the model because temperature is a mediator (*i.e*., a ‘pipe’). Second, and in contrast to the first, to estimate the total effect of temperature, year must be included in the model because it is a common cause (*i.e*., a ‘fork’). The inclusion of investigator era may affect the precision of coefficients (*e.g*., year, temperature), but should not bias the estimates because it is not linked to biodiversity through other unmeasured confounds. McElreath (2020) provides a clear introduction to forks, pipes, and the framework of causal inference; more details on DAGs and causal inference are described in Cinelli, Forney & Pearl (2024).

With our DAG in hand, we applied linear models to detect biodiversity change for four different metrics of biodiversity in the context of investigator effects. Then, we selected one biodiversity metric that changed over time and was insensitive to investigators, and tested whether it was related to ocean temperature. This step of attribution was intentional with respect to the chosen predictor (maximum sea surface temperature) and effect (Hill-Shannon diversity) and thus represents an *a priori* hypothesis test (rather than exploratory data analysis). For this reason, we did not test other metrics, or their relationship to other predictors.

### Detecting biodiversity change over time and across investigator eras

Temporal patterns were examined for richness, Hill-Shannon diversity, and Hill-Simpson diversity; these three diversity indices are known as Hill numbers (Hill, 1973). These three metrics are special cases of a single equation and are expressed on the same scale and in units of species (or unique taxa). What differs is the relative sensitivity of each metric to rare species, with sensitivity represented by the order *q*. When *q* = 0, rare and abundant species are treated equally; as *q* increases, so does the emphasis on abundant species. The use of three metrics (rather than just one) permits a more complete story about biodiversity change that incorporates nuances related to rare *vs*. abundant species, and their relative importance. Hill numbers can be interpreted as the effective number of unique species for all species (*q* = 0; richness), ‘common’ species (*q* = 1; Hill-Shannon), and ‘dominant’ species (*q* = 2; Hill-Simpson) (Chao et al., 2014). In contrast, species richness, the traditional Shannon index, and the Gini-Simpson index (all commonly used by ecologists) are not on the same scale, do not measure the same quantity, and cannot be compared directly (Roswell, Dushoff & Winfree, 2021). Lastly, we quantified the evenness of the taxa in the community, to separate the effects of richness *vs*. relative abundances of taxa (Heip, 1974; Chao & Ricotta, 2019). Hereafter, we will refer to all four of these measurements as ‘diversity metrics’; in addition we will refer to the number of taxa (species and genera) as ‘richness’, Hill-Shannon and Hill-Simpson indices as ‘diversity’.

Although the same 19 quadrats were sampled in each of the 18 years, the number of individuals sampled within quadrats was highly variable and could confound interpretations of changes in Hill numbers *per se* (Gotelli & Colwell, 2001). Therefore, we standardized samples at the site level using a coverage-based estimator. Coverage is a measure of how completely a community has been sampled, and better accounts for the underlying species abundance distribution of the community than traditional rarefaction (Hsieh, Ma & Chao, 2016; Roswell, Dushoff & Winfree, 2021).

We used a series of statistical models for inference and estimation of the linear trends in diversity metrics over the recent 30 years of the study (1993–2023). The models included the fixed effects of year and investigator era (Model 1; Appendix S2). We treated investigator era (*n* = 4) as a fixed effect in the model because eras are not exchangeable due to their ordered nature in time. These models were implemented in a Bayesian framework. The primary benefit of using a Bayesian approach was the ability to test whether biodiversity change occurred between the 1930s and the more recent sampling period (1993–2023); this is challenging because the historical baseline was from a single unreplicated transect. However, by simulating posterior predictions of diversity metrics, conditional on the models, we inferred strong evidence of change if the 95% prediction intervals from 1993 to 2023 did not envelope the historical baseline.

We incorporated weakly informative normal priors on the intercept and slope parameters; prior predictive distributions were visualized to ensure that a wide range of temporal trends were plausible. We fit the models with 3,000 iterations across four chains and discarded the first 1,500 iterations of each chain as warm-up, resulting in a posterior sample of 6,000 for each response. We inspected the chains for convergence, confirmed that the scale-reduction factor (*R*_*hat*_) was less than 1.05, and ensured that the minimum effect size (*n*_*eff*_) was greater than 1,000 for all parameters (Gelman et al., 2013). We used visual posterior predictive checks to assess model fit. All models were fit with Stan (Carpenter et al., 2017) using the R package “rethinking” (McElreath, 2020).

### Attribution of biodiversity change to temperature

Once we had estimated the effects of year and investigator era, we next wished to attribute the detected change over time to an ecologically relevant cause. Temperature is a fundamental physical factor that dictates biological rates from cellular reactions to species interactions (Kordas, Harley & O’Connor, 2011). Specifically, we chose maximum sea surface temperature because extreme, rather than average, metrics likely play a strong role in affecting ecological communities (Gaines & Denny, 1993); moreover, maximum (but not average) sea surface temperature increased by 0.01 °C yr^−1^ between 1938 and 2015 at HMS (Elahi, Miller & Litvin, 2020). We used the same hand-collected daily temperature time-series in this study, and quantified the maximum temperature as the 90^th^ percentile value for the year prior to the intertidal survey for a given year; in this way the hypothesized cause (temperature) precedes the effect (diversity) and thus strengthens the causal interpretation.

For this attribution step, we selected one diversity metric that exhibited change over time (a ‘year’ effect) and was least sensitive to investigator era. We applied three different models to examine the effect of temperature. First, we fit a naïve model that only considered the linear effect of temperature; the estimate of the temperature coefficient in this model is confounded by unmeasured factors covarying with year (Fig. 2). Second, we fit a model that included the linear effects of temperature, year, and era; this model accounted for potential confounds that covary linearly with year and the categorical effect of era (Model 2; Appendix S2). Third, we fit a Gaussian process model (Model 3; Appendix S2) that considered non-linear and autocorrelated confounds with year. By conditioning on the effect of year in the latter two models, we blocked the backdoor path to better estimate the direct causal influence of temperature on diversity. These models were implemented in a Bayesian framework, as described above for the detection of biodiversity change. For the Gaussian process model we used twice the number of iterations (12,000 across four chains); all parameter estimates had greater than 1000 effective samples except for the variance on the normal likelihood (n_eff_ = 962) but we considered this to be adequate because the chains were adequately mixed (*i.e*., *R*_*hat*_ = 1).

### Taxonomic change and geographic range

We next addressed the hypothesis that taxonomic change was associated with the geographic range of species. That is, we revisited the question of whether southern species on the Hewatt-Sagarin transect have increased in abundance relative to coastwide species, a pattern consistent with climate warming (Sagarin et al., 1999). We used the quantitative dataset for this analysis; taxa were removed if < 20 individuals were observed across the entire dataset, or if they occurred in fewer than five separate plots (10 individuals and five separate plots were the criteria used in Sagarin et al. (1999)). These inclusion criteria resulted in a dataset with 48 taxa comprising 99.7% of all the individuals counted across the study.

To summarize changes in the composition of animal taxa on the historical transect, we compared each year of sampling in the recent thirty years (1993–2023) to the historical baseline (1931–1933). First, the total abundance of each taxon (across 19 quadrats) was calculated for each year. Then the log change in abundance was estimated for each of the recent years, relative to the historical baseline (*log change* = *ln*[(*n*_*year*_ + 1)/(*n*_*baseline*_ + 1)]). The average log change and 95% confidence interval was calculated to summarize change in recent years relative to the historical baseline.

Geographic range assignments for these taxa were based on recent studies (Sanford et al., 2019; Micheli et al., 2020) and older taxonomic accounts (Morris, Abbott & Haderlie, 1980; Carlton, 2007). Species with a northern range limit south of Cape Mendocino, CA, were designated as ‘southern’. Species with a southern range limit north of Point Conception, CA, were designated as ‘northern’. Species with range limits extending beyond both of these points were designated as ‘coastwide’. None of the 48 taxa chosen for this analysis were categorized as ‘northern’. Two genera were assigned as uncertain.

We used the average log abundance for each taxon to test the effect of geographic range. Measurement error was incorporated into the statistical model to acknowledge the imperfect estimation of the average response; we did this by estimating a parameter for the unobserved ‘true’ log change using the standard error from the raw data (Model 4; Appendix S2). We calculated the posterior distribution of the difference between southern and coastwide species to explicitly test the effect of geographic range on relative change. This model was implemented in a Bayesian framework, as described above for the detection of biodiversity change, but with 2,000 iterations across four chains.

### Inference and scientific communication of results

To aid in the discussion of inferences from our statistical models we adopt a ‘language of evidence’ approach (Muff et al., 2022). This approach avoids binary decision making based on a single threshold (*e.g*., *P* = 0.05) and employs a more nuanced approach to communicate scientific results. We emphasize the use of credible intervals and their overlap (with zero, or intervals from different groups), and the probabilities of observing positive or negative point estimates (derived from posterior predictions). We consider non-overlapping 95% and 80% credible intervals to demonstrate ‘strong’ and ‘weak’ evidence of an effect; otherwise we infer no evidence for an effect. Similarly, slope estimates are considered to differ from zero with ‘strong’, ‘moderate’, and ‘weak’ evidence using thresholds of 95%, 90%, and 80% of posterior predictions, respectively.

### Software and data

We relied on the ‘tidyverse’ (Wickham et al., 2019), ‘iNext’ (Hsieh, Ma & Chao, 2016), and ‘patchwork’ (Pedersen, 2024) packages in R 4.3 (R Development Core Team, 2023) for data processing and visualization. All data and code are available in a permanent repository (https://purl.stanford.edu/fd733kb4905).

## Results

In all, 347,465 invertebrates were counted and identified. We checked our species names with those used by Hewatt (1937) and Barry et al. (1995), Sagarin et al. (1999). After harmonizing the taxonomy to the presently accepted names (Table S1), the database contained 185 unique taxa (143 species, 19 genera, 23 higher level clades), of which 119 species were observed in more than one era and thus could have been subject to a name change. Of these species, 43 species changed names once (36%), and 13 species changed names twice (11%). After processing the data for quantitative analysis, 327,415 individuals remained, comprised by 117 species and 17 genera. The average density of each taxon by investigator era is presented in Table S2 and details of the processing steps are provided in Table S3.

### Inferring biodiversity change across investigator eras

**Figure 3:**
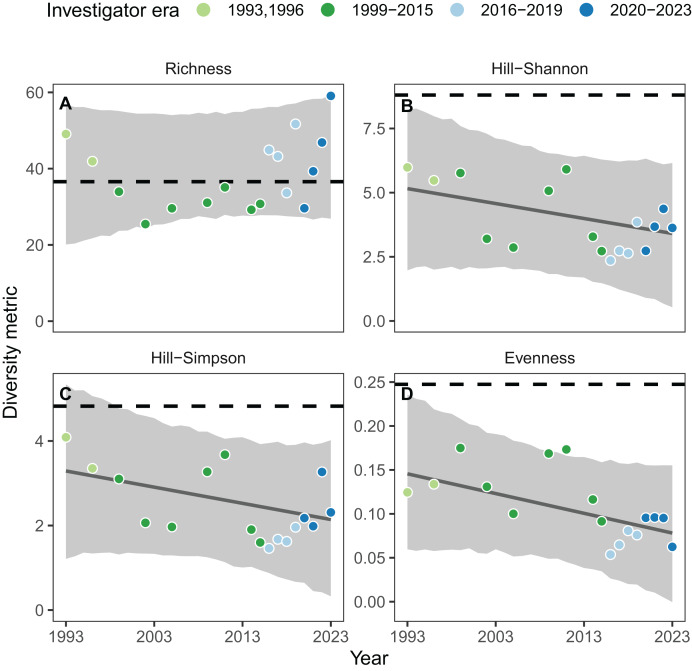
Biodiversity change of the animal community on the Hewatt-Sagarin transect over the recent thirty years (1993–2023) and relative to the initial survey in 1931–1933 (represented as dashed lines). There was no evidence for a linear trend in species richness (A), but weak evidence of negative trends in Hill-Shannon diversity (B), Hill-Simpson diversity (C), and species evenness (D). Solid lines represent the average effect of year on the response; shaded regions represent the 95% credible intervals for predictions of the diversity metrics. When the prediction intervals do not overlap the historical baseline in 1931–1933, we infer strong evidence of change over time. Note the different scales on the y-axis.

There was no evidence for change in the richness of the animal community on the Hewatt-Sagarin transect since the initial survey in 1931–1933, nor in the three recent decades (Fig. 3A). However, three other metrics of diversity showed weak evidence of decline over the recent three decades, and strong evidence of decline in the intervening six decades since the initial survey (Figs. 3B, 3C, 3D).

The temporal trend in richness was positive (1.6 taxa decade^−1^; 95% confidence interval (CI) [−5.5 to 8.8]) but highly uncertain (67% probability of increase). Hill-Shannon diversity declined weakly over the same period (Fig. 3B; −0.6 common taxa decade^−1^; 95% CI [−1.9 to −0.6]; 82% probability of decline). Hill-Simpson diversity exhibited a weak linear decline over the same period (Fig. 3C; −0.4 dominant taxa decade^−1^; 95% CI [−1.2 to 0.4]; 83% probability of decline). Evenness also exhibited a weak linear decline over the same period (Fig. 3D; −0.02 evenness decade^−1^; 95% CI [−0.06 to 0.01]; 89% probability of decline). Notably, there was strong evidence for change in Hill-Shannon diversity and evenness since the 1930s because the upper prediction intervals for these metrics did not overlap the historical baseline (Figs. 3B, 3D); strong evidence for change was observed for Hill-Simpson diversity after 1999 (Fig. 3C). On average, Hill-Shannon diversity declined by 41−61%, Hill-Simpson diversity declined by 32−56%, and evenness declined by 41−68% relative to the 1930s.

**Figure 4:**
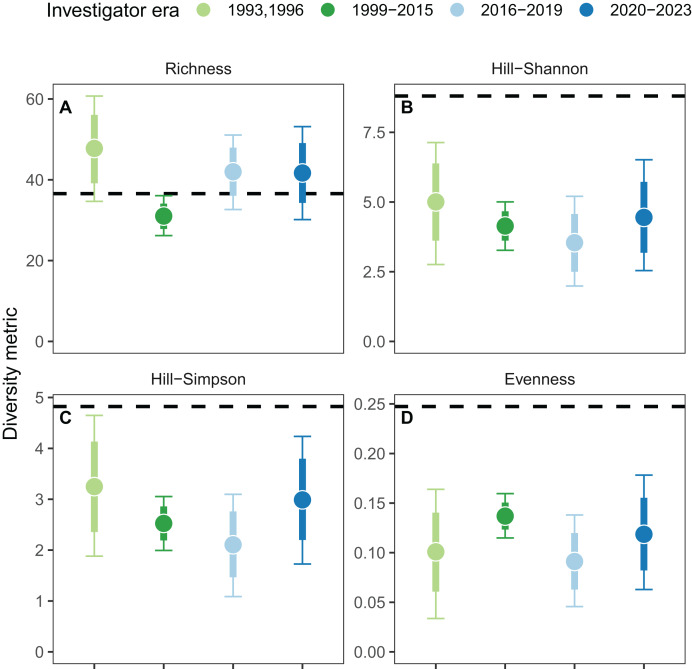
Biodiversity change of the animal community on the Hewatt-Sagarin transect over four recent investigator eras and relative to the initial survey in 1931–1933 (represented as dashed lines). There was weak evidence for an effect of era on species richness (A), but no evidence of an effect on Hill-Shannon diversity (B), Hill-Simpson diversity (C), or species evenness (D). Points represent model predictions for the effect of era on the average response; thick and thin error bars represent the 80 and 95% credible intervals for the average response, respectively. Note the different scales on the y-axis.

In addition to linear temporal trends, we simultaneously estimated the effects of investigator era and found weak evidence for richness, but not the other three diversity metrics (Fig. 4). Specifically, lower richness between 1999 and 2015 (Fig. 4A) coincided with one investigator era (1999–2015). During this era, species-accumulation curves saturated at relatively low values of site-scale richness (<50 taxa; Fig. S2) because fewer individuals were counted, particularly for mobile species (Fig. S3). Counts of sessile taxa exhibited boom and bust dynamics driven primarily by high barnacle densities. When split by mobility (Fig. S4), mobile taxa exhibited similar temporal trends to Fig. 3 (with all taxa) but sessile taxa demonstrated no temporal trends, indicating that most of the observed variation over time is due to mobile taxa.

**Figure 5:**
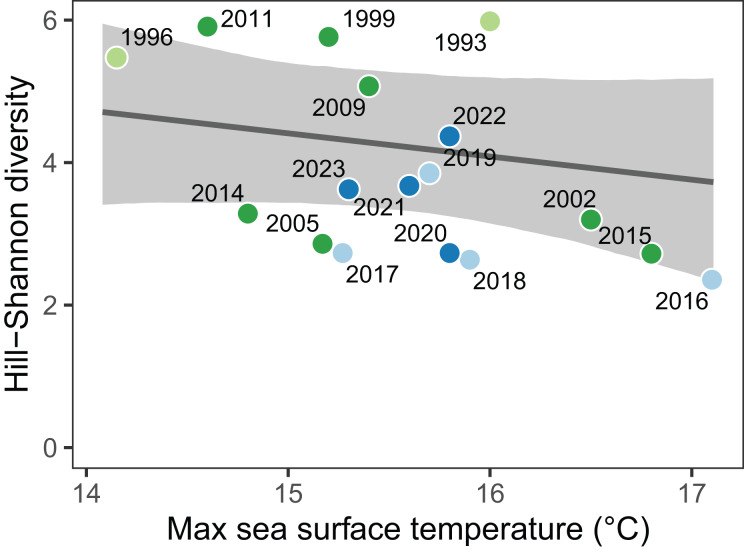
The diversity of the animal community on the Hewatt-Sagarin transect was negatively affected by maximum sea surface temperature. The causal estimate of temperature on Hill-Shannon diversity was weakly negative (−0.25 common taxa °C^−1^). Sea surface temperature was defined as the 90th percentile temperature in the year prior to sampling. Shaded region represents the 95% credible interval for the average effect of temperature on diversity. Point colors represent investigator eras as in Figs. 3 and 4.

A naïve regression of diversity against temperature indicated strong evidence for a negative association (−0.48 common taxa °C^−1^; 95% CI [−0.97 to 0.03]; 96.7% probability of a negative slope) but this effect is confounded by other potential drivers of change that are correlated with year (Fig. 2). The causal effect of maximum sea surface temperature on Hill-Shannon diversity, accounting for linear confounds that covaried with year and era, was moderately negative, with a slope of −0.34 common taxa °C^−1^ (95% CI [−0.82 to 0.15]; 91.9% probability of a negative slope). The causal effect of maximum sea surface temperature on Hill-Shannon diversity, accounting for non-linear and autocorrelated confounds with year (*i.e*., the Gaussian process model), was weakly negative, with a slope of −0.25 common taxa °C^−1^ (95% CI [−0.74 to 0.20]; 86% probability of a negative slope). We present the causal effect of temperature on diversity in Fig. 5, because we consider the Gaussian process model to be the most realistic (*i.e*., non-linear and autocorrelated) representation of potential confounds in time.

**Figure 6:**
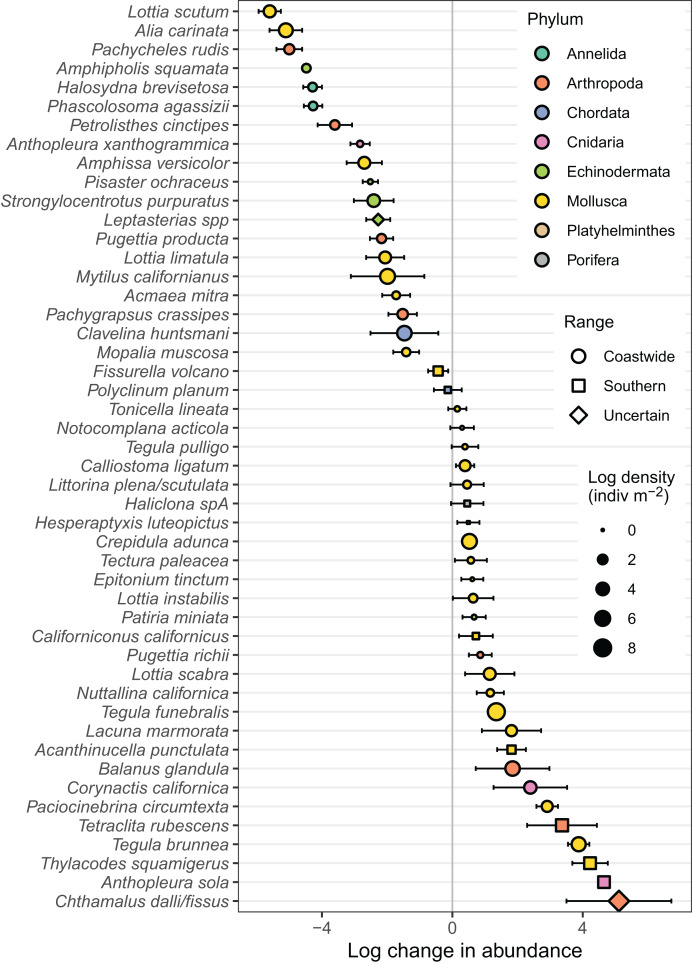
Changes in abundance of animal taxa on the Hewatt-Sagarin transect over the past 30 years (1993–2023) relative to the initial survey in 1931–1933. Change in abundance (the total abundance for each taxon in year *i*, where *i* ranges from 1993 to 2023) is expressed as the natural log of a ratio [ln(abundance + 1 in year *i*/abundance + 1 in the original survey)]. Points represent model predictions for the average response; error bars represent 95% confidence intervals. The size of points corresponds to the log mean density, averaged across all 18 years of sampling (1931–1933, 1993–2023). These 48 taxa comprise >99.7% of the individuals counted on the transect over the 90-year duration of the study.

**Figure 7:**
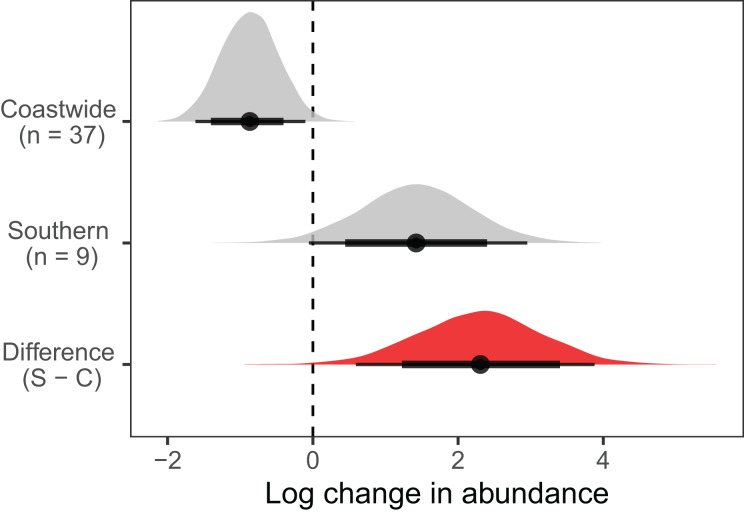
Geographic range affected the change in abundance of animal taxa on the Hewatt-Sagarin transect over the past 30 years (1993–2023) relative to the initial survey in 1931–1933. Average change in abundance was calculated relative to the initial survey in 1931–1933. The number of taxa in each range category is in parentheses; two taxa (*Chthamalus dalli/fissus* and *Leptasterias* spp.) whose ranges were uncertain were not included in the plot. The difference in average log change in abundance was calculated between southern and coastwide species. Points represent model predictions for the average response; thick and thin error bars represent the 80 and 95% credible intervals, respectively. Shaded areas represent the posterior distributions of the estimates.

The observed decline in Hill-Shannon diversity over time and with increasing sea surface temperature was accompanied by a preponderance of change in species composition relative to the historical baseline (Fig. 6, Table 1). Across 48 common taxa, 44 populations exhibited strong evidence of change relative to the historical baseline (*i.e*., the 95% confidence interval for the log response ratio did not overlap 0). Strong population declines were similarly common (*n* = 20) to strong increases (*n* = 22). The geographic range of species predicted variation in population change. On average, we observed declines in abundance of 37 coastwide species, but increases in abundance of nine southern species, relative to the historical baseline (Fig. 7). There was strong evidence for a difference between coastwide and southern species in the average response.

**Table 1:** Summary of observed animal abundances on the historical transect. Average densities (no. m^−2^; SD) of animal species are presented by investigator era, along with the log response ratio (LRR; 95% confidence interval in parentheses), representing the change in abundance over the recent 30 years (1993–2023) relative to the initial survey in 1931–1933. Change in abundance (the total abundance for each taxon in year *i*, where *i* ranges from 1993 to 2023) was calculated as the natural log of a ratio [ln(abundance + 1 in year *i*/abundance + 1 in the original survey)]. Range abbreviations are C (coastwide), S (southern), and U (uncertain). These 48 taxa comprise >99.7% of the individuals counted on the transect over the 90-year duration of the study. Note that 1931–1933 does not have a standard deviation because there was no temporal replication in that investigator era. See Fig. 6 for a visualization of LRR, and Table S2 for summarized abundances for all species.

Group	Species	Common name	Range	1931–1933	1993, 1996	1999–2015	2016–2019	2020–2023	LRR
Anthozoans	*Anthopleura sola*	Anemone	S	0	4.7 (0.3)	4.7 (0.7)	4.9 (0.2)	4.1 (1.1)	4.7 (4.6, 4.7)
Anthozoans	*Anthopleura xanthogrammica*	Anemone	C	1.1	0.2 (0)	0 (0)	0 (0)	0 (0.1)	−2.8 (−3.1, −2.5)
Anthozoans	*Corynactis californica*	Anemone	C	0	5.9 (0.1)	4.6 (4.2)	0 (0)	0 (0)	2.4 (1.3, 3.5)
Bivalves	*Mytilus californianus*	Mussel	C	9.3	0.3 (0.2)	0.2 (0.1)	3.2 (2.7)	87.2 (88.6)	−2 (−3.1, −0.9)
Crustaceans	*Balanus glandula*	Barnacle	C	0	121.3 (167.1)	0.3 (0.5)	0.9 (0.7)	0 (0)	1.8 (0.7, 3)
Crustaceans	*Chthamalus dalli/fissus*	Barnacle	U	0	254.1 (63.8)	122.6 (189.3)	2.3 (3)	129.8 (250.8)	5.1 (3.5, 6.7)
Crustaceans	*Pachycheles rudis*	Crab	C	8.5	0 (0)	0 (0)	0 (0)	0.3 (0.5)	−5 (-5.4, −4.6)
Crustaceans	*Pachygrapsus crassipes*	Crab	C	2.1	0.9 (0.2)	0.3 (0.3)	0.6 (0.4)	0.9 (0.1)	−1.5 (−2, −1.1)
Crustaceans	*Petrolisthes cinctipes*	Crab	C	3.7	0 (0)	0 (0.1)	0.1 (0.1)	0.6 (0.3)	−3.6 (−4.1, −3.1)
Crustaceans	*Pugettia producta*	Crab	C	1.8	0.5 (0.2)	0.2 (0.1)	0.2 (0.1)	0.2 (0.2)	−2.2 (−2.5, −1.8)
Crustaceans	*Pugettia richii*	Crab	C	0	0.3 (0.4)	0.1 (0.1)	0 (0.1)	0.1 (0.1)	0.9 (0.5, 1.2)
Crustaceans	*Tetraclita rubescens*	Barnacle	S	0	22.3 (23.4)	6.6 (10.2)	8.4 (15)	0 (0.1)	3.4 (2.3, 4.4)
Echinoderms	*Amphipholis squamata*	Brittle star	C	4.1	0 (0)	0 (0)	0 (0)	0 (0)	−4.5 (−4.6, −4.4)
Echinoderms	*Leptasterias* spp.	Sea star	U	1.9	0.8 (0.3)	0.1 (0.1)	0.1 (0)	0.2 (0.1)	−2.3 (−2.6, −1.9)
Echinoderms	*Patiria miniata*	Sea star	C	0	0.3 (0)	0 (0)	0.1 (0.1)	0 (0)	0.7 (0.3, 1)
Echinoderms	*Pisaster ochraceus*	Sea star	C	0.7	0 (0)	0 (0.1)	0 (0)	0 (0)	−2.5 (−2.8, −2.3)
Echinoderms	*Strongylocentrotus purpuratus*	Sea urchin	C	11.3	1.3 (0.5)	0.6 (0.7)	0.9 (0.9)	7.1 (5.6)	−2.4 (−3, −1.8)
Gastropods	*Acanthinucella punctulata*	Snail	S	0	0.5 (0.5)	0.2 (0.1)	0.2 (0.2)	0.8 (0.5)	1.8 (1.4, 2.3)
Gastropods	*Acmaea mitra*	Limpet	C	0.6	0.1 (0.1)	0.1 (0.1)	0.2 (0.1)	0.2 (0.4)	−1.7 (−2.2, −1.3)
Gastropods	*Alia carinata*	Snail	C	122.4	5 (1.6)	0.8 (0.5)	0.6 (0.8)	0.9 (1.1)	−5.1 (−5.6, −4.6)
Gastropods	*Amphissa versicolor*	Snail	C	9.1	2.2 (1.5)	0.2 (0.1)	0.6 (0.5)	3 (3.5)	−2.7 (−3.2, −2.2)
Gastropods	*Californiconus californicus*	Snail	S	0	0 (0)	0 (0)	0.1 (0.1)	0.5 (0.4)	0.7 (0.2, 1.2)
Gastropods	*Calliostoma ligatum*	Snail	C	0.5	1.2 (0.5)	1 (0.6)	0.9 (0.4)	0.4 (0.3)	0.4 (0.1, 0.7)
Gastropods	*Crepidula adunca*	Snail	C	11.3	19.5 (5.4)	19.6 (6.9)	22.1 (10.5)	20.6 (6.5)	0.5 (0.3, 0.7)
Gastropods	*Epitonium tinctum*	Snail	C	0	0.2 (0)	0 (0)	0 (0.1)	0.1 (0.1)	0.6 (0.3, 0.9)
Gastropods	*Fissurella volcano*	Keyhole limpet	S	0.7	1.7 (0.5)	0.4 (0.2)	0.5 (0.3)	0.4 (0.4)	−0.4 (−0.7, −0.1)
Gastropods	*Hesperaptyxis luteopictus*	Snail	S	0	0.1 (0)	0.1 (0.2)	0 (0)	0 (0)	0.5 (0.2, 0.8)
Gastropods	*Lacuna marmorata*	Snail	C	0	3.5 (1.6)	1.5 (1.4)	0 (0)	0 (0)	1.8 (0.9, 2.7)
Gastropods	*Littorina plena/scutulata*	Snail	C	0	0.3 (0.3)	0.1 (0.2)	0.1 (0.1)	0.4 (0.2)	0.5 (−0.1, 1)
Gastropods	*Lottia instabilis*	Limpet	C	0	0 (0)	0 (0)	1.1 (1)	0 (0)	0.6 (0, 1.3)
Gastropods	*Lottia limatula*	Limpet	C	5.5	1.5 (0.7)	0.3 (0.3)	3.5 (3.2)	1 (0.4)	−2.1 (−2.6, −1.5)
Gastropods	*Lottia scabra*	Limpet	C	0.1	1.7 (1.6)	2.9 (6)	1.4 (2.4)	0.4 (0.6)	1.1 (0.4, 1.9)
Gastropods	*Lottia scutum*	Limpet	C	33.1	0.2 (0.2)	0.2 (0.1)	0 (0)	0.1 (0.1)	−5.6 (−5.9, −5.3)
Gastropods	*Paciocinebrina circumtexta*	Snail	C	0	1 (0.2)	0.5 (0.3)	0.7 (0.2)	2.2 (0.6)	2.9 (2.6, 3.2)
Gastropods	*Tectura paleacea*	Limpet	C	0	0.7 (0.1)	0.1 (0.1)	0 (0)	0 (0)	0.6 (0.1, 1.1)
Gastropods	*Tegula brunnea*	Snail	C	0.1	30.6 (6)	7.5 (4.3)	9.1 (3)	8 (5.4)	3.9 (3.5, 4.2)
Gastropods	*Tegula funebralis*	Snail	C	47.1	150.5 (8.2)	124.5 (40.4)	271.7 (32.7)	286.5 (29.3)	1.3 (1.1, 1.6)
Gastropods	*Tegula pulligo*	Snail	C	0	0.5 (0.7)	0 (0)	0 (0)	0.1 (0.1)	0.4 (0, 0.8)
Gastropods	*Thylacodes squamigerus*	Snail	S	0	10.9 (5)	7.5 (5.1)	1.6 (1)	1.2 (0.9)	4.2 (3.7, 4.8)
Platyhelminths	*Notocomplana acticola*	Flatworm	C	0	0 (0)	0 (0)	0 (0)	0.2 (0.3)	0.3 (−0.1, 0.7)
Polychaetes	*Halosydna brevisetosa*	Segmented worm	C	4	0 (0)	0 (0)	0 (0)	0.1 (0.2)	−4.3 (−4.6, −4)
Polyplacophorans	*Mopalia muscosa*	Chiton	C	0.6	0.3 (0.1)	0 (0)	0.1 (0.1)	0.4 (0.3)	−1.4 (−1.8, −1)
Polyplacophorans	*Nuttallina californica*	Chiton	C	0	0.1 (0.2)	0.2 (0.2)	0.1 (0.2)	0.1 (0.1)	1.2 (0.7, 1.6)
Polyplacophorans	*Tonicella lineata*	Chiton	C	0	0.1 (0)	0 (0)	0.1 (0.1)	0.1 (0.1)	0.2 (−0.1, 0.4)
Poriferans	*Haliclona* sp. A	Sponge	S	0	0 (0)	0 (0)	0 (0)	0.5 (0.4)	0.5 (0, 1)
Sipunculids	*Phascolosoma agassizii*	Peanut worm	C	3.7	0 (0)	0 (0)	0 (0)	0.1 (0.2)	−4.3 (−4.6, −4)
Tunicates	*Clavelina huntsmani*	Sea squirt	C	17.3	12.4 (4.8)	3.9 (4.1)	16.6 (9.8)	31.8 (50.6)	−1.5 (−2.5, −0.4)
Tunicates	*Polyclinum planum*	Sea squirt	S	0.1	0.2 (0.2)	0.1 (0.1)	0.2 (0.3)	0.1 (0.3)	−0.1 (−0.6, 0.3)

## Discussion

Over nine decades, we have observed persistent and ongoing declines of animal diversity on a rocky intertidal shore in central California. We show that the loss of diversity was a consequence of population declines across many taxa and was associated strongly with warming seawater. Our study combines a historical baseline (Hewatt, 1937), a resurvey six decades later (Barry et al., 1995), and intermittent to annual monitoring over the subsequent three decades that spanned multiple investigators (Sagarin et al., 1999; Micheli et al., 2020). Therefore, we couched our inferences about temporal trends in biodiversity in the context of potential investigator effects.

One of the strengths of our intertidal sampling program is the enumeration of all macroscopic invertebrates in our quadrats. This feature permits the estimation of biodiversity metrics (*e.g*., richness, diversity), which rely on (i) quantifying abundance, and (ii) maintaining an ‘open’ species list, that is, a list that permits the addition of new species when discovered. Given the high density of intertidal invertebrates on the rocky shore, and the taxonomic expertise required to identify taxa, it is imperative to examine whether inferences on biodiversity change are sensitive to observer effects. In the context of sampling small and numerous rocky shore invertebrates, observers may vary in their taxonomic ability, eyesight, time constraints, attention to detail, and access to species identification references; these aspects can also change over time for the same observer. During each sampling event in our case study, the lead investigator may have enlisted the help of volunteers and students to assist with the identification and counting. Ultimately, it was the responsibility of each lead investigator to ensure quality control with respect to sampling and species identification, and thus we have focused on distinct investigator eras. We acknowledge that the actual observers add another layer of uncertainty to biodiversity estimates, but that is beyond the scope of the present study.

Our analyses suggest that investigator effects were particularly relevant for richness (*i.e*., the estimated number of taxa at a site). This metric hinges on the relative abundance of species and the identification of rare, cryptic and/or potentially new species. Richness is well known to be subject to biases related to sampling effort (Gotelli & Colwell, 2001), and richness was non-linearly associated with different investigator eras. The lack of a linear trend in richness in our study is consistent with theory suggesting a net balance between species colonizations and extinctions over time (Brown et al., 2001). Some recent syntheses emphasize no directional trends and high variability in local-scale biodiversity change (Vellend et al., 2013; Dornelas et al., 2014), but others that focused on temperate marine environments have demonstrated a trend of species gains (Elahi et al., 2015; Antão et al., 2020). Model projections of species richness in the temperate ocean support the notion of species gains on a large scale (Beaugrand et al., 2015; García Molinos et al., 2016), but longitudinal studies are needed to test these predictions.

In contrast to richness, diversity metrics (*i.e*., Hill-Shannon and Hill-Simpson) that reduce the leverage of rare species (Roswell, Dushoff & Winfree, 2021) were robust to investigator effects. The diversity of common (Hill-Shannon) and dominant (Hill-Simpson) taxa declined weakly over the past three decades but was clearly lower than the baseline established in the 1930s. On average, diversity declined by approximately 50% relative to the 1930s and was accompanied by a similar reduction in the evenness of animal taxa. These results are consistent with biodiversity loss and population decline on rocky shores observed in historical resurveys and long-term studies over the past two to five decades (Harley, 2011; Sorte et al., 2017; Starko et al., 2019; Petraitis & Dudgeon, 2020). Understanding the consequences of diversity loss for the structure and functioning of rocky shore communities remains an important challenge but will depend on species traits, interactions, and functional redundancy (Wootton & Emmerson, 2005; Bracken et al., 2008; Hillebrand & Matthiessen, 2009).

There were several taxonomic and functional changes associated with the decline in diversity. On average, southern species increased in abundance relative to coastwide species and thus the range-related population change first reported in the 1990s (Barry et al., 1995; Sagarin et al., 1999) has persisted over the past three decades. For example, southern sunburst anemones (*Anthopleura sola*) have become well-established on the transect, whereas the coastwide green anemone (*Anthopleura xanthogrammica*) has declined in abundance. The southern tube snail (*Thylacodes squamigerus*), first detected on the historical transect in the 1990s, is still present but at lower densities since 2015. Cone snails (*Californiconus californicus*), also of southern affinity, were first detected in 2016 and are still present in low density in the lower intertidal zone. These two species represent functionally unique gastropods on the transect, in that the tube snail is a sessile suspension feeder and the cone snail is a specialized predator (Morris, Abbott & Haderlie, 1980). The southern unicorn whelk (*Acanthinucella punctulata*) was first detected on the transect in 1993, and its densities have slowly increased over time; however, we have observed this species at Hopkins since 1974 and it was likely present prior to that (J. Watanabe, personal observation, 1974; Morris, Abbott & Haderlie, 1980). The southern volcano barnacle (*Tetraclita rubescens*) declined steadily since the 1990s, but new recruitment after 2023 has reestablished the population on the wave-exposed portion of the transect (R. Elahi, personal observation, 2023). The success of southern species on the historical transect is likely related to their physiological tolerance of warming temperatures, but only a few local species have been studied in this regard (Stillman & Somero, 2000; Somero, 2010).

Population change was also observed for many coastwide species. In particular, top snails (*Tegula brunnea* and especially *T. funebralis*) are now the most abundant molluscan grazers in the mid- to low-intertidal zones and their increase was associated with declines in predatory seastars (*Pisaster ochraceus, Leptasterias* spp.) (Paine, 1969; Gravem & Morgan, 2017). Similarly, the loss of *P. ochraceus* likely contributed to the dramatic increase of their mussel prey (*Mytilus californianus*) in 2020–2023 (Chapuis et al., 2026). Acorn barnacles (*Chthamalus* spp.), comprised by indistinguishable (in the field) northern and southern species (*C. dalli* and *fissus*, respectively) came to dominate some quadrats in the mid- to high-intertidal zone, which may have prompted an increase in the abundance of barnacle predators (*e.g*., the whelk, *Paciocinebrina circumtexta*). Limpets, in contrast, have declined across most species (*e.g*., *Lottia scutum, L. limatula, Acmaea mitra*) with the exception of *L. scabra*, a thermally tolerant species that lives high on the rocky shore (Dong et al., 2008). We speculate that the reduction in limpets is related to their morphology. Given their large foot and inability to retract within their shell, the body temperatures of limpets are more likely to match surrounding rock temperature which may influence survival during periods of thermal stress (Denny & Harley, 2006; Miller & Denny, 2011).

We also detected fluctuations in less conspicuous functional groups of species. Two gastropod detritivores (*Alia carinata, Amphissa versicolor*) and brittle stars (*Amphipholis squamata*) have declined since the 1930s. After prolonged decline, purple sea urchins (*Strongylocentrotus purpuratus*) have recovered to or exceeded historic levels; urchin recruits were particularly abundant in dense aggregations of mussel shells towards the wave exposed portion of the transect after 2020. An increase in the deposition of mussel shells and sand, associated with intense winter storms in 2022 and 2023, may have facilitated the recovery of urchins and other depleted cryptic species (*Pachycheles rudis, Petrolisthes cinctipes*, *Pugettia producta, Halosydna brevisetosa*, *Phascolosoma agassizi* and other polychaete worms) that occupy interstitial and mussel shell habitats. Distinguishing between a population rebound associated with an increase in favorable habitat *vs*. investigator effort remains a challenge for these cryptic and less common species.

The linear decline in Hill-Shannon diversity since the 1990s was associated strongly with increasing maximum sea surface temperature. After accounting for unmeasured effects that may have covaried with year (Fig. 2), we estimated the causal effect of temperature on diversity to be weakly negative (−0.25 common taxa °C^−1^). Although the estimated causal effect is an improvement over a naïve statistical association between diversity and temperature, our analysis still relies on a single time-series for inference; future work should emphasize replicated temperature and biodiversity time series for stronger inference of causal effects (Byrnes & Dee, 2025). Besides seawater temperature, many other physical factors that are relevant to the performance of intertidal organisms will covary with time (Gravem et al., 2024). We chose seawater temperature because it is important physiologically to marine animals, but also practical–it was the only physical covariate (within a close proximity to the historical transect) available since 1993. The body temperature of intertidal organisms will also be dictated by terrestrial conditions during low tide (Helmuth et al., 2002), and the physiological extremes imposed during emersion can dictate the survival and abundance of intertidal species (Miller, Harley & Denny, 2009).

Temporal change in the physical environment will affect intertidal animals directly *via* organismal performance, but also indirectly through changes in species interactions (Kordas, Harley & O’Connor, 2011; Kroeker & Sanford, 2022). In particular, the observed reduction in animal diversity may have been caused by the loss of large brown rockweeds (*Silvetia compressa*) (Sagarin et al., 1999). These seaweeds provide a large canopy that ameliorates thermal and desiccation stress and provides habitat to many invertebrates, particularly mobile taxa. Indeed, the biodiversity trends were driven primarily by mobile invertebrates, rather than sessile invertebrates. The latter were less speciose but exhibited large inter-annual population fluctuations, thus contributing to temporal variability but no directional trend in diversity indices. In contrast, mobile taxa exhibited strong linear declines in diversity. We found it surprising that mobile taxa exhibited a stronger signal of biodiversity change over time, considering that they can move in and out of quadrats and thus respond to the environmental conditions on any given sampling day. However, this source of variation may be smaller than that induced by the extreme recruitment potential of sessile barnacles and mussels. The potential consequences of an algal canopy on animal diversity highlights a weakness of the historical program, which does not presently quantify macroalgae in the field. We began photographing the quadrats in 2015 (Elahi & Watanabe, 2023) and this will allow us to test hypotheses about temporal change in seaweeds and associated changes in the community.

Another limitation of this long-term study is that the animals were sampled from contiguous quadrats along a single transect that may not be representative of other rocky shores in the region. We view this as a worthwhile tradeoff with the duration of the study and the level of taxonomic detail. Such trade-offs in design and implementation are common in other long-term rocky shore studies (Kaplanis, 2023). As a stark contrast to our study, a spatially extensive, well-replicated, and coordinated effort across the west coast of North America must, out of necessity, limit surveys to a pre-selected group of focal species; more thorough taxonomic surveys can only be completed on a less regular basis when funding permits (Multi-Agency Rocky Intertidal Network; Gilbane et al., 2022). Our historical resurvey with its open species list highlights the value of marine stations that enable otherwise unfundable monitoring efforts at small spatial scales but fine taxonomic resolution and long duration.

We see room for improvement in our approach to the historical study that does not necessarily require more effort, which would be untenable without sustained dedicated funding. Instead, a reallocation of current effort would permit stronger inferences about the patterns and underlying causes of future biodiversity change. For example, by focusing on a subset of the existing quadrats, and selecting new permanent quadrats stratified along the vertical intertidal gradient, we could better achieve representation of the local rocky shore. Increasing the numbers of photoquadrats on the rocky shore would allow the documentation of changes to functional groups of sessile invertebrates and seaweeds with stronger replication but minimal field effort. Many of the trade-offs in sampling design are well known (Murray, Ambrose & Dethier, 2006), but implementing new strategies in the midst of a historical survey will require care and explicit documentation.

There are several recommendations from our intertidal sampling program that are relevant to inferences about biodiversity change from long-term ecological monitoring studies. First, it is important to have an open species list to monitor new colonizers, especially in the context of climate change and species range shifts (Sagarin et al., 1999; Sanford et al., 2019). Second, for monitoring studies that span different investigators, statistical analyses should consider the effect of investigator era. Third, consistency in the number of samples (*e.g*., quadrats) does not guarantee consistency in species abundances, which we interpret here as variation in sampling effort by investigators. Therefore, inferences about trends in species richness can be sensitive to investigator effects because of the inherent difficulty in observing and identifying rare or cryptic species. In contrast, diversity indices that deemphasize rare species may be more reliable indicators of biodiversity change in long-term studies that change hands. Fourth, when possible, lead investigators should train the next generation in the field to provide practical advice and discuss the nuances associated with sampling. In lieu of this best-case scenario, the finer details of sampling should be documented to minimize investigator effects.

## Supplemental Information

10.7717/peerj.21099/supp-1 Supplemental Information 1 Species names used in this article, by Sagarin and coauthors (Barry et al., 1995, Sagarin et al., 1999), and by (Hewatt, 1937v). The ‘Analysis’ column indicates the taxon used in this study for analyses of biodiversity change. NA indicates that the taxon was not recorded.

10.7717/peerj.21099/supp-2 Supplemental Information 2 Summary of observed animal abundances on the historical transect. Average densities (no. m ^-2^; SD) of animal species are presented by investigator era. Note that 1931-1933 does not have a standard deviation because there was no temporal replication in that investigator era.

10.7717/peerj.21099/supp-3 Supplemental Information 3 List of processing steps and associated observations (*i.e*., total abundance) to prepare the quantitative dataset for analysis.

10.7717/peerj.21099/supp-4 Supplemental Information 4 Visualization of the relative positions, tidal heights (m relative to mean lower low water), and vertical relief (m) of quadrats along the Hewatt-Sagarin transect. The historical transect begins at the uppermost limits of the rocky shore and is set perpendicular to the shoreline. Nearshore quadrats are separated from farshore quadrats by a channel at minus tides. Square symbols represent ’core’ quadrats that were resurveyed from 1993 to 2023 and diamonds represent ’extra’ quadrats that were resurveyed from 2020 to 2023; small dots represent quadrats that have not been resurveyed but for which we have tidal height and vertical relief data. Vertical relief was measured as the maximum vertical distance within a quadrat. The historical data archived by (Hewatt, 1937) included quadrats 11-100, 105.

10.7717/peerj.21099/supp-5 Supplemental Information 5 Species-accumulation curves from the repeated sampling conducted on the Hewatt-Sagarin transect during five investigator eras. The observed richness is plotted as a point; lines before the point represent rarefaction and lines beyond the point represent extrapolation. Shaded areas represent 95% confidence intervals.

10.7717/peerj.21099/supp-6 Supplemental Information 6 Total number of individuals from the repeated sampling conducted on the Hewatt-Sagarin transect, separated by mobility (mobile *vs* sessile animals).

10.7717/peerj.21099/supp-7 Supplemental Information 7 Temporal trends in site-scale alpha diversity metrics for the animal community on the Hewatt-Sagarin transect, separated by all taxa, mobile taxa, and sessile taxa. Diversity metrics were estimated using a coverage-based estimator (estimate ± 95% confidence intervals). Note that panels for all taxa are duplicated from Fig. 3A-C from the main text.

10.7717/peerj.21099/supp-8 Supplemental Information 8 Description of survey methods for the Hewatt-Sagarin transect, Hopkins Marine Station, California, USA.

10.7717/peerj.21099/supp-9 Supplemental Information 9 Statistical models.

## Additional Information and Declarations

### Data Availability

The following information was supplied regarding data availability:

The Raw data and code are available at The Stanford Digital Repository:

- Elahi, R., Micheli, F., and Watanabe, J. (2026). Persistent loss of animal diversity on a rocky shore over nine decades and across multiple investigators (data and code). Stanford Digital Repository. Available at https://purl.stanford.edu/fd733kb4905. https://doi.org/10.25740/fd733kb4905.

## References

[ref-1] Antão LH, Bates AE, Blowes SA, Waldock C, Supp SR, Magurran AE, Dornelas M, Schipper AM (2020). Temperature-related biodiversity change across temperate marine and terrestrial systems. Nature Ecology & Evolution.

[ref-2] Arif S, MacNeil MA (2022). Predictive models aren’t for causal inference. Ecology Letters.

[ref-3] Barry JP, Baxter CH, Sagarin RD, Gilman SE (1995). Climate-related, long-term faunal changes in a California rocky intertidal community. Science.

[ref-4] Beaugrand G, Edwards M, Raybaud V, Goberville E, Kirby RR (2015). Future vulnerability of marine biodiversity compared with contemporary and past changes. Nature Climate Change.

[ref-5] Benedetti-Cechi L, Airoldi L, Abbiati M, Cinelli F (1996). Estimating the abundance of benthic invertebrates: a comparison of procedures and variability between observers. Marine Ecology Progress Series.

[ref-6] Bracken MES, Friberg SE, Gonzalez-Dorantes CA, Williams SL (2008). Functional consequences of realistic biodiversity changes in a marine ecosystem. Proceedings of the National Academy of Sciences USA.

[ref-7] Brown JH, Ernest SKM, Parody JM, Haskell JP (2001). Regulation of diversity: maintenance of species richness in changing environments. Oecologia.

[ref-8] Byrnes JE, Dee LE (2025). Causal inference with observational data and unobserved confounding variables. Ecology Letters.

[ref-9] Carlton JT (2007). The Light and Smith manual: intertidal invertebrates from central California to Oregon.

[ref-10] Carpenter B, Gelman A, Hoffman MD, Lee D, Goodrich B, Betancourt M, Brubaker M, Guo J, Li P, Riddell A (2017). Stan: a probabilistic programming language. Journal of Statistical Software.

[ref-11] Chao A, Gotelli NJ, Hsieh T, Sander EL, Ma K, Colwell RK, Ellison AM (2014). Rarefaction and extrapolation with Hill numbers: a framework for sampling and estimation in species diversity studies. Ecological Monographs.

[ref-12] Chao A, Ricotta C (2019). Quantifying evenness and linking it to diversity, beta diversity, and similarity. Ecology.

[ref-13] Chapuis M, McDevitt-Irwin J, Palmisciano M, Elahi R, Detrait A, Gorum O, Vogel C, Chui E, Haupt A, Micheli F (2026). Recent changes in mussel beds following mass mortality of a keystone marine predator. Marine Ecology Progress Series.

[ref-14] Cinelli C, Forney A, Pearl J (2024). A crash course in good and bad controls. Sociological Methods & Research.

[ref-15] Dee LE, Ferraro PJ, Severen CN, Kimmel KA, Borer ET, Byrnes JEK, Clark AT, Hautier Y, Hector A, Raynaud X, Reich PB, Wright AJ, Arnillas CA, Davies KF, MacDougall A, Mori AS, Smith MD, Adler PB, Bakker JD, Brauman KA, Cowles J, Komatsu K, Knops JMH, McCulley RL, Moore JL, Morgan JW, Ohlert T, Power SA, Sullivan LL, Stevens C, Loreau M (2023). Clarifying the effect of biodiversity on productivity in natural ecosystems with longitudinal data and methods for causal inference. Nature Communications.

[ref-16] Denny MW, Harley CD (2006). Hot limpets: predicting body temperature in a conductance-mediated thermal system. Journal of Experimental Biology.

[ref-17] Dong Y, Miller LP, Sanders JG, Somero GN (2008). Heat-shock protein 70 (Hsp70) expression in four limpets of the genus Lottia: interspecific variation in constitutive and inducible synthesis correlates with in situ exposure to heat stress. The Biological Bulletin.

[ref-18] Dornelas M, Gotelli NJ, McGill B, Shimadzu H, Moyes F, Sievers C, Magurran AE (2014). Assemblage time series reveal biodiversity change but not systematic loss. Science.

[ref-19] Douda J, Doudová J, Holeštová A, Chudomelová M, Vild O, Boublík K, Černá M, Havrdová A, Petřík P, Pychová N, Smyčková M, Šebesta J, Vaníček J, Hédl R (2023). Historical sampling error: a neglected factor in long-term biodiversity change research. Biological Conservation.

[ref-20] Durden JM, Bett BJ, Schoening T, Morris KJ, Nattkemper TW, Ruhl HA (2016). Comparison of image annotation data generated by multiple investigators for benthic ecology. Marine Ecology Progress Series.

[ref-21] Elahi R, Micheli F, Watanabe JM (2024). Passing the quadrat: inferring biodiversity change over time and across investigators.

[ref-22] Elahi R, Miller LP, Litvin SY (2020). Historical comparisons of body size are sensitive to data availability and ecological context. Ecology.

[ref-23] Elahi R, O’Connor MI, Byrnes JE, Dunic J, Eriksson BK, Hensel MJS, Kearns PJ (2015). Recent trends in local-scale marine biodiversity reflect community structure and human impacts. Current Biology.

[ref-24] Elahi R, Watanabe JM (2023). Hewatt transect photoquadrats. Stanford Digital Repository. https://purl.stanford.edu/nr783rs2739.

[ref-25] Gaines SD, Denny MW (1993). The largest, smallest, highest, lowest, longest, and shortest: extremes in ecology. Ecology.

[ref-26] García Molinos J, Halpern BS, Schoeman DS, Brown CJ, Kiessling W, Moore PJ, Pandolfi JM, Poloczanska ES, Richardson AJ, Burrows MT (2016). Climate velocity and the future global redistribution of marine biodiversity. Nature Climate Change.

[ref-27] Gelman A, Carlin JB, Stern HS, Dunson DB, Vehtari A, Rubin DB (2013). Bayesian Data Analysis.

[ref-28] Gilbane L, Ambrose RF, Burnaford JL, Helix ME, Miner CM, Murray S, Sullivan KM, Whitaker SG (2022). Long-term sustainability of ecological monitoring: perspectives from the Multi-Agency Rocky Intertidal Network. Partnerships in Marine Research.

[ref-29] Gonzalez A, Chase JM, O’Connor MI (2023). A framework for the detection and attribution of biodiversity change. Philosophical Transactions of the Royal Society B.

[ref-30] Gotelli NJ, Colwell RK (2001). Quantifying biodiversity: procedures and pitfalls in the measurement and comparison of species richness. Ecology Letters.

[ref-31] Gravem SA, Morgan SG (2017). Shifts in intertidal zonation and refuge use by prey after mass mortalities of two predators. Ecology.

[ref-32] Gravem SA, Poirson BN, Robinson JW, Menge BA (2024). Resistance of rocky intertidal communities to oceanic climate fluctuations. PLOS ONE.

[ref-33] Harley CDG (2011). Climate change, keystone predation, and biodiversity loss. Science.

[ref-34] Heip C (1974). A new index measuring evenness. Journal of the Marine Biological Association of the United Kingdom.

[ref-35] Helmuth B, Harley CD, Halpin PM, O’donnell M, Hofmann GE, Blanchette CA (2002). Climate change and latitudinal patterns of intertidal thermal stress. Science.

[ref-36] Hewatt WG (1934). Ecological studies on selected marine intertidal communities of Monterey Bay. Ph.D. Thesis. Stanford University of Stanford, California.

[ref-37] Hewatt WG (1937). Ecological studies on selected marine intertidal communities of Monterey Bay, California. American Midland Naturalist.

[ref-38] Hill MO (1973). Diversity and evenness: a unifying notation and its consequences. Ecology.

[ref-39] Hillebrand H, Matthiessen B (2009). Biodiversity in a complex world: consolidation and progress in functional biodiversity research. Ecology Letters.

[ref-40] Hsieh T, Ma KH, Chao A (2016). iNEXT: an R package for rarefaction and extrapolation of species diversity (Hill numbers). Methods in Ecology and Evolution.

[ref-41] Hughes BB, Beas-Luna R, Barner AK, Brewitt K, Brumbaugh DR, Cerny-Chipman EB, Close SL, Coblentz KE, de Nesnera KL, Drobnitch ST, Figurski JD, Focht B, Friedman M, Freiwald J, Heady KK, Heady WN, Hettinger A, Johnson A, Karr KA, Mahoney B, Moritsch MM, Osterback A-MK, Reimer J, Robinson J, Rohrer T, Rose JM, Sabal M, Segui LM, Shen C, Sullivan J, Zuercher R, Raimondi PT, Menge BA, Grorud-Colvert K, Novak M, Carr MH (2017). Long-term studies contribute disproportionately to ecology and policy. BioScience.

[ref-42] Kaplanis NJ (2023). Insight into best practices: a review of long-term monitoring of the rocky intertidal zone of the Northeast Pacific Coast. Frontiers in Marine Science.

[ref-43] Kordas RL, Harley CDG, O’Connor MI (2011). Community ecology in a warming world: the influence of temperature on interspecific interactions in marine systems. Journal of Experimental Marine Biology and Ecology.

[ref-44] Kroeker KJ, Sanford E (2022). Ecological leverage points: species interactions amplify the physiological effects of global environmental change in the ocean. Annual Review of Marine Science.

[ref-45] Light SF (1941). A laboratory and field textbook of invertebrate zoology.

[ref-46] Magurran AE (2021). Measuring biological diversity. Current Biology.

[ref-47] Magurran AE, Baillie SR, Buckland ST, Dick JM, Elston DA, Scott EM, Smith RI, Somerfield PJ, Watt AD (2010). Long-term datasets in biodiversity research and monitoring: assessing change in ecological communities through time. Trends in Ecology & Evolution.

[ref-48] McElreath R (2020). Statistical Rethinking: A Bayesian Course with Examples in R and Stan.

[ref-49] Micheli F, Carlton J, Pearse JS, Selgrath J, Elahi R, Watanabe JM, Mach ME, McDevitt-Irwin J, Pearse VB, N B, Baxter CH (2020). Field stations as sentinels of change. Frontiers in Ecology and the Environment.

[ref-50] Miller LP, Denny MW (2011). Importance of behavior and morphological traits for controlling body temperature in littorinid snails. The Biological Bulletin.

[ref-51] Miller LP, Harley CD, Denny MW (2009). The role of temperature and desiccation stress in limiting the local-scale distribution of the owl limpet, *Lottia gigantea*. Functional Ecology.

[ref-52] Montràs-Janer T, Suggitt AJ, Fox R, Jönsson M, Martay B, Roy DB, Walker KJ, Auffret AG (2024). Anthropogenic climate and land-use change drive short-and long-term biodiversity shifts across taxa. Nature Ecology & Evolution.

[ref-53] Morris R, Abbott D, Haderlie E (1980). Intertidal Invertebrates of California.

[ref-54] Morrison LW (2016). Observer error in vegetation surveys: a review. Journal of Plant Ecology.

[ref-55] Muff S, Nilsen EB, O’Hara RB, Nater CR (2022). Rewriting results sections in the language of evidence. Trends in Ecology & Evolution.

[ref-56] Murray SN, Ambrose R, Dethier MN (2006). Monitoring Rocky Shores.

[ref-58] Paine RT (1969). The *Pisaster-Tegula* interaction: prey patches, predator food preference, and intertidal community structure. Ecology.

[ref-59] Pearl J (2010). An introduction to causal inference. The International Journal of Biostatistics.

[ref-60] Pedersen TL (2024). Patchwork: The composer of plots.

[ref-61] Pereira HM, Navarro LM, Martins IS (2012). Global biodiversity change: the bad, the good, and the unknown. Annual Review of Environment and Resources.

[ref-62] Petraitis PS, Dudgeon S (2020). Declines over the last two decades of five intertidal invertebrate species in the western North Atlantic. Communications Biology.

[ref-63] Provin BF (1949). Populations of marine animals on a permanent transect at Mussel Point. https://purl.stanford.edu/qp336xx5959.

[ref-64] R Development Core Team (2023). R: a language and environment for statistical computing.

[ref-65] Richardson A, Walne A, John A, Jonas T, Lindley J, Sims D, Stevens D, Witt M (2006). Using continuous plankton recorder data. Progress in Oceanography.

[ref-66] Ricketts EF, Calvin J (1948). Between Pacific Tides.

[ref-67] Rinella MJ, Strong DJ, Vermeire LT (2020). Omitted variable bias in studies of plant interactions. Ecology.

[ref-68] Roswell M, Dushoff J, Winfree R (2021). A conceptual guide to measuring species diversity. Oikos.

[ref-69] Sagarin RD, Barry JP, Gilman SE, Baxter CH (1999). Climate-related change in an intertidal community over short and long time scales. Ecological Monographs.

[ref-70] Sagarin R, Pauchard A (2010). Observational approaches in ecology open new ground in a changing world. Frontiers in Ecology and the Environment.

[ref-71] Sanford E, Sones JL, García-Reyes M, Goddard JH, Largier JL (2019). Widespread shifts in the coastal biota of northern California during the 2014–2016 marine heatwaves. Scientific Reports.

[ref-72] Schrodt F, Beck M, Estopinan J, Bowler DE, Fontaine C, Gaüzère P, Goury R, Grenié M, Martins IS, Morueta-Holme N, Santini L, Hedde M, Martin G, Porcher E, Si-Moussi S, Tzivanopoulos M, Vernham G, Violle C, Thuiller W (2025). Advancing causal inference in ecology: pathways for biodiversity change detection and attribution. Methods in Ecology and Evolution.

[ref-73] Siegel K, Dee LE (2025). Foundations and future directions for causal inference in ecological research. Ecology Letters.

[ref-74] Silvertown J, Poulton P, Johnston E, Edwards G, Heard M, Biss PM (2006). The Park Grass Experiment 1856–2006: its contribution to ecology. Journal of Ecology.

[ref-75] Smith RI, Carlton JT (1975). Light’s manual: intertidal invertebrates of the central California coast.

[ref-76] Somero G (2010). The physiology of climate change: how potentials for acclimatization and genetic adaptation will determine ‘winners’ and ‘losers’. Journal of Experimental Biology.

[ref-77] Sorte CJ, Davidson VE, Franklin MC, Benes KM, Doellman MM, Etter RJ, Hannigan RE, Lubchenco J, Menge BA (2017). Long-term declines in an intertidal foundation species parallel shifts in community composition. Global Change Biology.

[ref-78] Southward A, Hawkins S, Burrows M (1995). Seventy years’ observations of changes in distribution and abundance of zooplankton and intertidal organisms in the western English Channel in relation to rising sea temperature. Journal of Thermal Biology.

[ref-79] Starko S, Bailey LA, Creviston E, James KA, Warren A, Brophy MK, Danasel A, Fass MP, Townsend JA, Neufeld CJ (2019). Environmental heterogeneity mediates scale-dependent declines in kelp diversity on intertidal rocky shores. PLOS ONE.

[ref-80] Stillman JH, Somero GN (2000). A comparative analysis of the upper thermal tolerance limits of eastern Pacific porcelain crabs, genus Petrolisthes: influences of latitude, vertical zonation, acclimation, and phylogeny. Physiological and Biochemical Zoology.

[ref-81] Suggitt AJ, Lister DG, Thomas CD (2019). Widespread effects of climate change on local plant diversity. Current Biology.

[ref-82] Sunday JM, Bates AE, Dulvy NK (2012). Thermal tolerance and the global redistribution of animals. Nature Climate Change.

[ref-83] Vellend M (2016). The Theory of Ecological Communities (MPB-57).

[ref-84] Vellend M, Baeten L, Myers-Smith IH, Elmendorf SC, Beauséjour R, Brown CD, DeFrenne P, Verheyen K, Wipf S (2013). Global meta-analysis reveals no net change in local-scale plant biodiversity over time. Proceedings of the National Academy of Sciences, USA.

[ref-85] Wickham H, Averick M, Bryan J, Chang W, McGowan LD, François R, Grolemund G, Hayes A, Henry L, Hester J, Kuhn M, Pedersen TL, Miller E, Bache SM, Müller K, Ooms J, Robinson D, Seidel DP, Spinu V, Takahashi K, Vaughan D, Wilke C, Woo K, Yutani H (2019). Welcome to the tidyverse. Journal of Open Source Software.

[ref-86] Wootton JT, Emmerson M (2005). Measurement of interaction strength in nature. Annual Review of Ecology Evolution and Systematics.

[ref-87] WoRMS Editorial Board (2023). World register of marine species (WoRMS). https://www.marinespecies.org.

