# Supplementary material for: Persistent loss of animal diversity on a rocky shore over nine decades and across multiple investigators
Source: PeerJ. 2026 Apr 16;14:e21099. doi: 10.7717/peerj.21099 (PMC13092228; doi:10.7717/peerj.21099)
Supplement: Supplemental Information 1 — The ‘Analysis’ column indicates the taxon used in this study for analyses of biodiversity change. NA indicates that the taxon was not recorded. [file peerj-14-21099-s001.docx]

**Table S1: Table of species names used in this paper, by Sagarin and coauthors (Barry et al. 1995, Sagarin et al. 1999), and by Hewatt (1937).** The ‘Analysis’ column indicates the taxon used in this study for analyses of biodiversity change. NA indicates that the taxon was not recorded.

| **Phylum** | **Taxon** | **Taxon (Sagarin)** | **Taxon (Hewatt)** | **Analysis** |
| --- | --- | --- | --- | --- |
| Annelida | *Arabella iricolor* | *Arabella iricolor* | *-* | species |
| Annelida | *Cirriformia spirabrancha* | *-* | *-* | species |
| Annelida | *Drilonereis spp* | *-* | *-* | genus |
| Annelida | *Errantia clade* | *-* | *-* | subclass |
| Annelida | *Eteone pacifica* | *-* | *-* | species |
| Annelida | *Flabesymbios commensalis* | *-* | *-* | species |
| Annelida | *Glycera americana* | *Glycera americana* | *-* | species |
| Annelida | *Halosydna brevisetosa* | *Halosydna insignis* | *Halosydna insignis* | species |
| Annelida | *Hyboscolex pacificus* | *-* | *-* | species |
| Annelida | *Leitoscoloplos pugettensis* | *-* | *-* | species |
| Annelida | *Lumbrineridae clade* | *-* | *-* | family |
| Annelida | *Lumbrineridae spA* | *-* | *-* | family |
| Annelida | *Lumbrineridae spB* | *-* | *-* | family |
| Annelida | *Lumbrineridae spC* | *-* | *-* | family |
| Annelida | *Lumbrineris spp* | *Lumbrinereis spp* | *Lumbrinereis sp* | family |
| Annelida | *Marphysa sanguinea* | *-* | *-* | species |
| Annelida | *Nereis grubei* | *-* | *-* | genus |
| Annelida | *Nereis spp* | *Nereis spp* | *Nereis sp* | genus |
| Annelida | *Nereis vexillosa* | *-* | *-* | genus |
| Annelida | *Notomastus spp* | *-* | *-* | genus |
| Annelida | *Oxydromus pugettensis* | *-* | *-* | species |
| Annelida | *Paraonides spp* | *-* | *-* | genus |
| Annelida | *Phascolosoma agassizii* | *Phascolosoma agassizii* | *Physcasoma agassizi* | species |
| Annelida | *Sedentaria clade* | *-* | *-* | subclass |
| Annelida | *Serpula columbiana* | *-* | *Serpula columbiana* | genus |
| Annelida | *Serpula spp* | *-* | *-* | genus |
| Annelida | *Serpula vermicularis* | *-* | *-* | genus |
| Annelida | *Serpulidae clade* | *-* | *-* | subclass |
| Annelida | *Sipuncula clade* | *-* | *-* | order |
| Annelida | *Spirorbis spp* | *Spirorbis spp* | *Spirorbis sp* | genus |
| Annelida | *Thelepus crispus* | *-* | *-* | species |
| Arthropoda | *Amphipoda clade* | *-* | *-* | order |
| Arthropoda | *Ampithoe spp* | *-* | *-* | genus |
| Arthropoda | *Atylopsis spp* | *Atylopsis spp* | *Atylopsis sp* | genus |
| Arthropoda | *Balanomorpha clade* | *-* | *-* | order |
| Arthropoda | *Balanus glandula* | *Balanus glandula* | *Balanus glandula* | species |
| Arthropoda | *Betaeus longidactylus* | *-* | *-* | species |
| Arthropoda | *Brachyura clade* | *-* | *-* | order |
| Arthropoda | *Cancer productus* | *Cancer productus* | *Cancer productus* | species |
| Arthropoda | *Cancridae clade* | *-* | *-* | family |
| Arthropoda | *Chthamalus spp* | *Chthamalus spp* | *-* | genus |
| Arthropoda | *Cirolana harfordi* | *Cirolana harfordi* | *Cirolana harfordi* | species |
| Arthropoda | *Cryptolithodes sitchensis* | *Cryptolithodes sitchensis* | *-* | species |
| Arthropoda | *Hemigrapsus nudus* | *Hemigrapsus nudus* | *Hemigrapsus nudus* | species |
| Arthropoda | *Heptacarpus sitchensis* | *Heptacarpus pictus* | *Hippolyte californiensis* | species |
| Arthropoda | *Idotea spp* | *-* | *-* | genus |
| Arthropoda | *Idotea urotoma* | *Idotea urotoma* | *Idothea rectilinea* | genus |
| Arthropoda | *Isopoda clade* | *-* | *-* | order |
| Arthropoda | *Ligia occidentialis* | *Ligia occidentialis* | *Ligyda occidentalis* | genus |
| Arthropoda | *Lophopanopeus bellus* | *-* | *-* | genus |
| Arthropoda | *Lophopanopeus heathii* | *Lophopanopeus heathii* | *Lophopanopeus heathii* | genus |
| Arthropoda | *Lophopanopeus leucomanus* | *Lophopanopeus leucomanus* | *-* | genus |
| Arthropoda | *Lophopanopeus spp* | *-* | *-* | genus |
| Arthropoda | *Loxorhynchus crispatus* | *Loxorhynchus crispatus* | *-* | species |
| Arthropoda | *Megabalanus californicus* | *Megabalanus californicus* | *Balanus tintinnabulum californicus* | species |
| Arthropoda | *Melita palmata* | *Melita palmata* | *Melita palmata* | genus |
| Arthropoda | *Pachycheles rudis* | *Pachycheles rudis* | *Pachycheles rudis* | species |
| Arthropoda | *Pachygrapsus crassipes* | *Pachygrapsus crassipes* | *Pachygrapsus crassipes* | species |
| Arthropoda | *Pagurus spp* | *Pagurus spp* | *-* | genus |
| Arthropoda | *Paraxanthias taylori* | *Paraxanthias taylori* | *Xanthias taylori* | species |
| Arthropoda | *Petrolisthes cinctipes* | *Petrolisthes cinctipes* | *Petrolisthes cinctipes* | species |
| Arthropoda | *Petrolisthes eriomerus* | *-* | *-* | species |
| Arthropoda | *Petrolisthes spp* | *-* | *-* | genus |
| Arthropoda | *Pinnotheridae clade* | *-* | *-* | family |
| Arthropoda | *Pollicipes polymerus* | *Pollicipes polymerus* | *Mitella polymerus* | species |
| Arthropoda | *Pugettia foliata* | *Mimulus foliatus* | *Mimulus foliatus* | species |
| Arthropoda | *Pugettia producta* | *Pugettia producta* | *Pugettia productus* | species |
| Arthropoda | *Pugettia richii* | *Pugettia richii* | *Pugettia richii* | species |
| Arthropoda | *Pugettia spp* | *-* | *-* | genus |
| Arthropoda | *Pycnogonida clade* | *-* | *-* | family |
| Arthropoda | *Pycnogonum stearnsi* | *-* | *-* | family |
| Arthropoda | *Romaleon antennarium* | *Cancer antennarius* | *Cancer antennarius* | species |
| Arthropoda | *Romaleon jordani* | *Cancer jordani* | *Cancer jordani* | species |
| Arthropoda | *Spirontocaris picta* | *Spirontocaris picta* | *Spirontocaris picta* | genus |
| Arthropoda | *Tetraclita rubescens* | *Tetraclita rubescens* | *Tetraclita squamosa rubescens* | species |
| Bryozoa | *Bryozoa clade* | *-* | *-* | phylum |
| Bryozoa | *Bugula neritina* | *-* | *-* | species |
| Bryozoa | *Cryptosula pallasiana* | *Cryptosula pallasiana* | *Hippodiplosia poliasiana* | species |
| Bryozoa | *Integripelta bilabiata* | *-* | *-* | species |
| Bryozoa | *Watersipora subtorquata* | *-* | *-* | species |
| Chordata | *Anoplarchus purpurescens* | *-* | *-* | species |
| Chordata | *Aplidium californicum* | *Aplidium californicum* | *Amaroucium californicum* | species |
| Chordata | *Aplidium solidum* | *-* | *-* | species |
| Chordata | *Aplidium spp* | *-* | *-* | genus |
| Chordata | *Ascidiacea clade* | *-* | *-* | class |
| Chordata | *Clavelina huntsmani* | *Clavelina huntsmani* | *Clavelina sp* | species |
| Chordata | *Cottidae clade* | *-* | *-* | family |
| Chordata | *Distaplia occidentalis* | *-* | *-* | species |
| Chordata | *Eudistoma diaphanes* | *-* | *-* | species |
| Chordata | *Eudistoma molle* | *Archidistoma molle* | *-* | species |
| Chordata | *Eudistoma psammion* | *-* | *-* | species |
| Chordata | *Gibbonsia montereyensis* | *-* | *-* | species |
| Chordata | *Oligocottus maculosus* | *Oligocottus maculosus* | *-* | species |
| Chordata | *Perophora annectens* | *-* | *-* | species |
| Chordata | *Polyclinum planum* | *Polyclinum planum* | *Glossophorum planum* | species |
| Chordata | *Pycnoclavella stanleyi* | *-* | *-* | species |
| Chordata | *Rimicola eigenmanni* | *Rimicola eigenmanni* | *-* | species |
| Chordata | *Synoicum spp* | *-* | *-* | genus |
| Cnidaria | *Abietinaria spp* | *-* | *-* | genus |
| Cnidaria | *Aglaophenia spp* | *-* | *-* | genus |
| Cnidaria | *Aglaophenia struthionides* | *-* | *Aglaophenia struthionides* | genus |
| Cnidaria | *Anthopleura artemisia* | *-* | *-* | genus |
| Cnidaria | *Anthopleura elegantissima* | *Anthopleura elegantissima clonal* | *Cribrina elegantissima* | species |
| Cnidaria | *Anthopleura sola* | *Anthopleura elegantissima solitary* | *-* | species |
| Cnidaria | *Anthopleura xanthogrammica* | *Anthopleura xanthogrammica* | *Cribrina xanthogrammica* | species |
| Cnidaria | *Corynactis californica* | *Corynactis californica* | *-* | species |
| Cnidaria | *Epiactis prolifera* | *Epiactis prolifera* | *-* | species |
| Cnidaria | *Eudendrium spp* | *-* | *-* | genus |
| Cnidaria | *Symplectoscyphus spp* | *-* | *Sertularia pulchella* | genus |
| Echinodermata | *Amphiodia occidentalis* | *Amphiodia occidentalis* | *Amphiodia occidentalis* | species |
| Echinodermata | *Amphipholis squamata* | *Amphipholis pugetana* | *Amphipholis pugetana* | species |
| Echinodermata | *Asteroidea clade* | *-* | *-* | class |
| Echinodermata | *Henricia leviuscula* | *Henricia leviuscula* | *-* | species |
| Echinodermata | *Leptasterias spp* | *Leptasterias spp* | *Leptasterias aequalis* | genus |
| Echinodermata | *Leptosynapta albicans* | *Leptosynapta inhaerens* | *Leptosynapta inhaerens* | species |
| Echinodermata | *Lissothuria nutriens* | *Lissothuria nutriens* | *Thyonepsolus nutriens* | species |
| Echinodermata | *Ophiactis simplex* | *Ophiactis simplex* | *-* | species |
| Echinodermata | *Ophioderma panamense* | *Ophioderma panamense* | *-* | species |
| Echinodermata | *Ophiothrix spiculata* | *Ophiothrix spiculata* | *Ophiothrix spiculata* | species |
| Echinodermata | *Ophiuroidea clade* | *-* | *-* | class |
| Echinodermata | *Patiria miniata* | *Asterina miniata* | *Patiria miniata* | species |
| Echinodermata | *Pisaster ochraceus* | *Pisaster ochraceus* | *Pisaster ochraceus* | species |
| Echinodermata | *Strongylocentrotus purpuratus* | *Strongylocentrotus purpuratus* | *Strongylocentrotus purpuratus* | species |
| Mollusca | *Acanthinucella punctulata* | *Acanthina punctulata* | *Acanthina lapilloides* | species |
| Mollusca | *Acmaea mitra* | *Lottia mitra* | *Acmaea mitra* | species |
| Mollusca | *Alia carinata* | *Mitrella carinata* | *Columbella carinata* | species |
| Mollusca | *Amphissa columbiana* | *Amphissa columbiana* | *-* | species |
| Mollusca | *Amphissa versicolor* | *Amphissa versicolor* | *Amphissa versicolor* | species |
| Mollusca | *Antisabia panamensis* | *Hipponix cranioides* | *Hipponix antiquatus* | species |
| Mollusca | *Aplysia californica* | *-* | *-* | species |
| Mollusca | *Atrimitra idae* | *-* | *-* | species |
| Mollusca | *Bivalvia clade* | *-* | *-* | class |
| Mollusca | *Californiconus californicus* | *-* | *-* | species |
| Mollusca | *Calliostoma annulatum* | *-* | *-* | species |
| Mollusca | *Calliostoma canaliculatum* | *Calliostoma canaliculatum* | *-* | species |
| Mollusca | *Calliostoma ligatum* | *Calliostoma ligatum* | *Calliostoma costatum* | species |
| Mollusca | *Calliostoma spp* | *-* | *-* | genus |
| Mollusca | *Ceratodoris rosacea* | *Hopkinsia rosacea* | *Hopkinsia rosacea* | species |
| Mollusca | *Ceratostoma foliatum* | *Ceratostoma foliatum* | *-* | species |
| Mollusca | *Chaetopleura gemma* | *Chaetopleura gemma* | *-* | species |
| Mollusca | *Chama pellucida* | *Chama pellucida* | *Chama pellucida* | species |
| Mollusca | *Chlamys hastata* | *-* | *-* | species |
| Mollusca | *Coryphella spp* | *-* | *-* | genus |
| Mollusca | *Coryphella trilineata* | *Coryphella trilineata* | *-* | species |
| Mollusca | *Crassadoma gigantea* | *Hinnites giganteus* | *-* | species |
| Mollusca | *Crepidula adunca* | *Crepidula adunca* | *Crepidula adunca* | species |
| Mollusca | *Cyanoplax hartwegii* | *Cyanoplax hartwegii* | *Lepidochitona hartwegii* | species |
| Mollusca | *Diaphoreolis lagunae* | *Cuthona lagunae* | *-* | species |
| Mollusca | *Diaulula sandiegensis* | *-* | *-* | species |
| Mollusca | *Diodora aspera* | *-* | *-* | species |
| Mollusca | *Dirona spp* | *-* | *-* | genus |
| Mollusca | *Doridina clade* | *-* | *-* | order |
| Mollusca | *Doriopsilla albopunctata* | *Doriopsilla albopunctata* | *-* | species |
| Mollusca | *Epitonium indianorum* | *-* | *-* | species |
| Mollusca | *Epitonium tinctum* | *Epitonium tinctum* | *-* | species |
| Mollusca | *Eulithidium pulloides* | *-* | *-* | genus |
| Mollusca | *Fissurella volcano* | *Fissurella volcano* | *Fissurella volcano* | species |
| Mollusca | *Fissurellidea bimaculata* | *Megatebennus bimaculatus* | *-* | species |
| Mollusca | *Gari californica* | *-* | *-* | species |
| Mollusca | *Gastropoda clade* | *-* | *-* | class |
| Mollusca | *Geitodoris heathi* | *-* | *Discodoris heathi* | species |
| Mollusca | *Gigahomalopoma luridum* | *Homalopoma luridum* | *Leptothyra carpenteri* | species |
| Mollusca | *Haliotis cracherodii* | *Haliotis cracherodii* | *-* | species |
| Mollusca | *Hermissenda crassicornis* | *Hermissenda crassicornis* | *Hermissenda crassicornis* | genus |
| Mollusca | *Hermissenda opalescens* | *-* | *-* | genus |
| Mollusca | *Hermissenda spp* | *-* | *-* | genus |
| Mollusca | *Hesperaptyxis luteopictus* | *Fusinus luteopictus* | *-* | species |
| Mollusca | *Hespererato vitellina* | *Erato vitellina* | *-* | species |
| Mollusca | *Kelletia kelletii* | *-* | *-* | species |
| Mollusca | *Kellia laperousii* | *Kellia laperousii* | *Kellia laperousii* | species |
| Mollusca | *Lacuna marmorata* | *Lacuna marmorata* | *-* | species |
| Mollusca | *Lacuna porrecta* | *-* | *-* | species |
| Mollusca | *Lacuna unifasciata* | *-* | *-* | species |
| Mollusca | *Lepidozona cooperi* | *-* | *-* | genus |
| Mollusca | *Lepidozona mertensii* | *Lepidozona mertensii* | *-* | genus |
| Mollusca | *Limacia cockerelli* | *Laila cockerelli* | *Laila cockerelli* | species |
| Mollusca | *Lirobittium spp* | *-* | *-* | genus |
| Mollusca | *Littorina keenae* | *Littorina keenae* | *Littorina planaxis* | species |
| Mollusca | *Littorina scutulata* | *Littorina scutulata* | *Littorina scutulata* | species |
| Mollusca | *Lottia alaska* | *-* | *-* | genus |
| Mollusca | *Lottia asmi* | *Lottia asmi* | *Acmaea asmi* | species |
| Mollusca | *Lottia digitalis* | *Lottia digitalis* | *Acmaea digitalis* | species |
| Mollusca | *Lottia fenestrata* | *-* | *-* | genus |
| Mollusca | *Lottia instabilis* | *-* | *-* | species |
| Mollusca | *Lottia limatula* | *Lottia limatula* | *Acmaea limatula* | species |
| Mollusca | *Lottia paradigitalis* | *Lottia paradigitalis* | *-* | species |
| Mollusca | *Lottia pelta* | *Lottia pelta* | *Acmaea cassis pelta hybrid* | genus |
| Mollusca | *Lottia persona* | *-* | *-* | genus |
| Mollusca | *Lottia scabra* | *Macclintockia scabra* | *Acmaea scabra* | species |
| Mollusca | *Lottia scutum* | *Tectura scutum* | *Acmaea patina* | species |
| Mollusca | *Lottia spp* | *-* | *-* | genus |
| Mollusca | *Margarites salmoneus* | *-* | *-* | species |
| Mollusca | *Mitrella tuberosa* | *-* | *-* | species |
| Mollusca | *Modiolus carpenteri* | *Modiolus carpenteri* | *-* | genus |
| Mollusca | *Mopalia ciliata* | *-* | *-* | genus |
| Mollusca | *Mopalia lignosa* | *-* | *-* | genus |
| Mollusca | *Mopalia muscosa* | *Mopalia muscosa* | *Mopalia muscosa* | species |
| Mollusca | *Mopalia spp* | *-* | *-* | genus |
| Mollusca | *Muricidae clade* | *-* | *-* | family |
| Mollusca | *Mytilisepta bifurcata* | *-* | *-* | species |
| Mollusca | *Mytilus californianus* | *Mytilus californianus* | *Mytilus californianus* | species |
| Mollusca | *Mytilus spp* | *Mytilus edulis* | *-* | genus |
| Mollusca | *Nassarius mendicus* | *Nassarius mendicus* | *-* | species |
| Mollusca | *Neostylidium eschrichtii* | *Bittium eschrichtii* | *-* | species |
| Mollusca | *Nucella emarginata* | *Nucella emarginata* | *Thais emarginata* | genus |
| Mollusca | *Nucella lamellosa* | *-* | *-* | genus |
| Mollusca | *Nucella spp* | *-* | *-* | genus |
| Mollusca | *Nudibranchia clade* | *-* | *-* | order |
| Mollusca | *Nuttallina californica* | *Nuttallina californica* | *Nuttallina californica* | species |
| Mollusca | *Odetta fetella* | *-* | *-* | species |
| Mollusca | *Onchidella carpenteri* | *Onchidella borealis* | *-* | species |
| Mollusca | *Paciocinebrina atropurpurea* | *-* | *-* | genus |
| Mollusca | *Paciocinebrina circumtexta* | *Ocenebra circumtexta* | *-* | species |
| Mollusca | *Paciocinebrina interfossa* | *-* | *Tritonalia interfossa* | genus |
| Mollusca | *Paciocinebrina lurida* | *Ocenebra lurida* | *Tritonalia lurida* | genus |
| Mollusca | *Paciocinebrina spp* | *-* | *-* | genus |
| Mollusca | *Paciocinebrina subangulata* | *-* | *-* | genus |
| Mollusca | *Petaloconchus montereyensis* | *-* | *-* | species |
| Mollusca | *Polyplacophora clade* | *-* | *-* | class |
| Mollusca | *Pseudochama exogyra* | *-* | *-* | species |
| Mollusca | *Pseudomelatoma torosa* | *Pseudomelatoma torosa* | *-* | species |
| Mollusca | *Pseudopusula californiana* | *-* | *-* | species |
| Mollusca | *Rostanga pulchra* | *Rostanga pulchra* | *Rostanga pulchra* | species |
| Mollusca | *Tectura paleacea* | *Tectura paleacea* | *-* | species |
| Mollusca | *Tegula brunnea* | *Tegula brunnea* | *Tegula brunnea* | species |
| Mollusca | *Tegula funebralis* | *Tegula funebralis* | *Tegula funebralis* | species |
| Mollusca | *Tegula montereyi* | *Tegula montereyi* | *-* | species |
| Mollusca | *Tegula pulligo* | *Tegula pulligo* | *-* | species |
| Mollusca | *Thylacodes squamigerus* | *Serpulorbis squamigerus* | *-* | species |
| Mollusca | *Tonicella lineata* | *Tonicella lineata* | *Lepidochitona lineata* | species |
| Mollusca | *Triopha catalinae* | *-* | *Triopha carpenteri* | species |
| Mollusca | *Urosalpinx cinerea* | *-* | *-* | species |
| Nemertea | *Emplectonema gracile* | *Emplectonema gracile* | *-* | species |
| Nemertea | *Nemertea clade* | *-* | *-* | phylum |
| Nemertea | *Paranemertes peregrina* | *Paranemertes peregrina* | *Paranemertes peregrina* | species |
| Platyhelminthes | *Hylocelis californica* | *Alloioplana californica* | *Planocera californica* | species |
| Platyhelminthes | *Notocomplana acticola* | *-* | *-* | species |
| Platyhelminthes | *Platyhelminthes clade* | *-* | *-* | phylum |
| Porifera | *Antho karykina* | *Plocamia karykina* | *Plocamia karykinos* | species |
| Porifera | *Clathria pennata* | *-* | *-* | species |
| Porifera | *Haliclona spA* | *-* | *-* | species |
| Porifera | *Porifera clade* | *-* | *-* | phylum |
