# Supplementary material for: Persistent loss of animal diversity on a rocky shore over nine decades and across multiple investigators
Source: PeerJ. 2026 Apr 16;14:e21099. doi: 10.7717/peerj.21099 (PMC13092228; doi:10.7717/peerj.21099)
Supplement: Supplemental Information 2 — Average densities (no. m -2; SD) of animal species are presented by investigator era. Note that 1931-1933 does not have a standard deviation because there was no temporal replication in that investigator era. [file peerj-14-21099-s002.docx]

**Table S2: Summary of observed animal abundances on the historical transect.** Average densities (no. m^-2^; SD) of animal species are presented by investigator era. Note that 1931-1933 does not have a standard deviation because there was no temporal replication in that investigator era.

| **Taxon** | **Phylum** | **Group** | **1931-1933** | **1993, 1996** | **1999-2015** | **2016-2019** | **2020-2023** |
| --- | --- | --- | --- | --- | --- | --- | --- |
| *Arabella iricolor* | Annelida | Polychaetes | 0 | 0.4 (0.2) | 0 (0) | 0 (0) | 0 (0) |
| *Cirriformia spirabrancha* | Annelida | Polychaetes | 0 | 0 (0) | 0 (0) | 0 (0) | 0 (0) |
| *Drilonereis spp* | Annelida | Polychaetes | 0 | 0 (0) | 0 (0) | 0 (0) | 0 (0) |
| *Glycera americana* | Annelida | Polychaetes | 0 | 0.1 (0.1) | 0 (0) | 0 (0) | 0 (0) |
| *Halosydna brevisetosa* | Annelida | Polychaetes | 4 | 0 (0) | 0 (0) | 0 (0) | 0.1 (0.2) |
| *Hyboscolex pacificus* | Annelida | Polychaetes | 0 | 0 (0) | 0 (0) | 0 (0) | 0 (0) |
| *Leitoscoloplos pugettensis* | Annelida | Polychaetes | 0 | 0 (0) | 0 (0) | 0 (0) | 0.2 (0.4) |
| *Marphysa sanguinea* | Annelida | Polychaetes | 0 | 0 (0) | 0 (0) | 0 (0) | 0 (0) |
| *Nereis spp* | Annelida | Polychaetes | 0 | 0 (0) | 0 (0) | 0 (0) | 0 (0) |
| *Oxydromus pugettensis* | Annelida | Polychaetes | 0 | 0 (0) | 0 (0) | 0 (0) | 0 (0) |
| *Phascolosoma agassizii* | Annelida | Sipunculids | 3.7 | 0 (0) | 0 (0) | 0 (0) | 0.1 (0.2) |
| *Serpula spp* | Annelida | Polychaetes | 0 | 0.1 (0.1) | 0 (0) | 0 (0) | 0 (0) |
| *Balanus glandula* | Arthropoda | Crustaceans | 0 | 121.3 (167.1) | 0.3 (0.5) | 0.9 (0.7) | 0 (0) |
| *Cancer productus* | Arthropoda | Crustaceans | 0.2 | 0 (0) | 0 (0) | 0 (0.1) | 0 (0) |
| *Chthamalus dalli/fissus* | Arthropoda | Crustaceans | 0 | 254.1 (63.8) | 122.6 (189.3) | 2.3 (3) | 129.8 (250.8) |
| *Cryptolithodes sitchensis* | Arthropoda | Crustaceans | 0 | 0 (0) | 0 (0) | 0 (0) | 0 (0) |
| *Hemigrapsus nudus* | Arthropoda | Crustaceans | 0.2 | 0 (0) | 0 (0) | 0.1 (0.1) | 0 (0.1) |
| *Idotea spp* | Arthropoda | Crustaceans | 0 | 0 (0) | 0 (0) | 0 (0) | 0 (0) |
| *Ligia spp* | Arthropoda | Crustaceans | 0 | 0 (0) | 0 (0) | 0 (0) | 0 (0) |
| *Lophopanopeus spp* | Arthropoda | Crustaceans | 0 | 0.1 (0) | 0 (0) | 0 (0) | 0 (0) |
| *Loxorhynchus crispatus* | Arthropoda | Crustaceans | 0 | 0 (0) | 0 (0) | 0 (0) | 0 (0) |
| *Megabalanus californicus* | Arthropoda | Crustaceans | 0.9 | 0 (0) | 0 (0) | 0 (0) | 0 (0) |
| *Pachycheles rudis* | Arthropoda | Crustaceans | 8.5 | 0 (0) | 0 (0) | 0 (0) | 0.3 (0.5) |
| *Pachygrapsus crassipes* | Arthropoda | Crustaceans | 2.1 | 0.9 (0.2) | 0.3 (0.3) | 0.6 (0.4) | 0.9 (0.1) |
| *Paraxanthias taylori* | Arthropoda | Crustaceans | 0 | 0.1 (0.1) | 0 (0) | 0 (0) | 0 (0) |
| *Petrolisthes cinctipes* | Arthropoda | Crustaceans | 3.7 | 0 (0) | 0 (0.1) | 0.1 (0.1) | 0.6 (0.3) |
| *Petrolisthes eriomerus* | Arthropoda | Crustaceans | 0 | 0 (0) | 0 (0) | 0 (0) | 0.3 (0.4) |
| *Pollicipes polymerus* | Arthropoda | Crustaceans | 0 | 0 (0) | 0 (0) | 0 (0) | 0 (0) |
| *Pugettia foliata* | Arthropoda | Crustaceans | 0 | 0 (0) | 0 (0) | 0 (0) | 0 (0) |
| *Pugettia producta* | Arthropoda | Crustaceans | 1.8 | 0.5 (0.2) | 0.2 (0.1) | 0.2 (0.1) | 0.2 (0.2) |
| *Pugettia richii* | Arthropoda | Crustaceans | 0 | 0.3 (0.4) | 0.1 (0.1) | 0 (0.1) | 0.1 (0.1) |
| *Romaleon antennarium* | Arthropoda | Crustaceans | 0.1 | 0 (0) | 0 (0) | 0 (0) | 0 (0) |
| *Romaleon jordani* | Arthropoda | Crustaceans | 0 | 0 (0) | 0 (0) | 0 (0) | 0.1 (0.2) |
| *Tetraclita rubescens* | Arthropoda | Crustaceans | 0 | 22.3 (23.4) | 6.6 (10.2) | 8.4 (15) | 0 (0.1) |
| *Integripelta bilabiata* | Bryozoa | Bryozoans | 0 | 0 (0) | 0 (0) | 0 (0) | 0 (0) |
| *Watersipora subtorquata* | Bryozoa | Bryozoans | 0 | 0 (0) | 0 (0) | 0 (0) | 0 (0) |
| *Aplidium solidum* | Chordata | Ascidians | 0 | 0 (0) | 0 (0) | 0 (0) | 0.3 (0.4) |
| *Clavelina huntsmani* | Chordata | Ascidians | 17.3 | 12.4 (4.8) | 3.9 (4.1) | 16.6 (9.8) | 31.8 (50.6) |
| *Distaplia occidentalis* | Chordata | Ascidians | 0 | 0 (0) | 0 (0) | 0 (0) | 0 (0) |
| *Eudistoma diaphanes* | Chordata | Ascidians | 0 | 0 (0) | 0 (0) | 0 (0) | 0.2 (0.3) |
| *Eudistoma molle* | Chordata | Ascidians | 0 | 0.2 (0.2) | 0 (0) | 0 (0) | 0.5 (0.5) |
| *Eudistoma psammion* | Chordata | Ascidians | 0 | 0 (0) | 0 (0) | 0.1 (0.2) | 0.1 (0.1) |
| *Perophora annectens* | Chordata | Ascidians | 0 | 0 (0) | 0 (0) | 0 (0) | 0 (0) |
| *Polyclinum planum* | Chordata | Ascidians | 0.1 | 0.2 (0.2) | 0.1 (0.1) | 0.2 (0.3) | 0.1 (0.3) |
| *Pycnoclavella stanleyi* | Chordata | Ascidians | 0 | 0 (0) | 0 (0) | 0 (0) | 0 (0) |
| *Synoicum spp* | Chordata | Ascidians | 0 | 0 (0) | 0 (0) | 0 (0) | 0 (0) |
| *Anoplarchus purpurescens* | Chordata | Fishes | 0 | 0 (0) | 0 (0) | 0 (0) | 0 (0.1) |
| *Gibbonsia montereyensis* | Chordata | Fishes | 0 | 0 (0) | 0 (0) | 0 (0) | 0 (0) |
| *Rimicola eigenmanni* | Chordata | Fishes | 0 | 0 (0) | 0 (0) | 0 (0) | 0 (0) |
| *Anthopleura elegantissima* | Cnidaria | Anthozoans | 0.4 | 0 (0) | 0 (0) | 0.1 (0.1) | 0 (0) |
| *Anthopleura sola* | Cnidaria | Anthozoans | 0 | 4.7 (0.3) | 4.7 (0.7) | 4.9 (0.2) | 4.1 (1.1) |
| *Anthopleura xanthogrammica* | Cnidaria | Anthozoans | 1.1 | 0.2 (0) | 0 (0) | 0 (0) | 0 (0.1) |
| *Corynactis californica* | Cnidaria | Anthozoans | 0 | 5.9 (0.1) | 4.6 (4.2) | 0 (0) | 0 (0) |
| *Abietinaria spp* | Cnidaria | Hydroids | 0 | 0 (0) | 0 (0) | 0 (0) | 0 (0.1) |
| *Aglaophenia spp* | Cnidaria | Hydroids | 0 | 0 (0) | 0 (0) | 0.1 (0.1) | 0 (0.1) |
| *Eudendrium spp* | Cnidaria | Hydroids | 0 | 0 (0) | 0 (0) | 0 (0.1) | 0 (0) |
| *Amphiodia occidentalis* | Echinodermata | Echinoderms | 0 | 0 (0) | 0 (0) | 0 (0) | 0.3 (0.7) |
| *Amphipholis squamata* | Echinodermata | Echinoderms | 4.1 | 0 (0) | 0 (0) | 0 (0) | 0 (0) |
| *Leptasterias spp* | Echinodermata | Echinoderms | 1.9 | 0.8 (0.3) | 0.1 (0.1) | 0.1 (0) | 0.2 (0.1) |
| *Leptosynapta albicans* | Echinodermata | Echinoderms | 0.2 | 0 (0) | 0 (0) | 0 (0) | 0 (0.1) |
| *Lissothuria nutriens* | Echinodermata | Echinoderms | 0 | 0 (0) | 0 (0) | 0 (0) | 0 (0) |
| *Ophiactis simplex* | Echinodermata | Echinoderms | 0 | 0 (0) | 0 (0) | 0 (0) | 0 (0) |
| *Ophioderma panamense* | Echinodermata | Echinoderms | 0 | 0 (0) | 0 (0) | 0 (0) | 0 (0) |
| *Ophiothrix spiculata* | Echinodermata | Echinoderms | 0 | 0 (0) | 0 (0) | 0 (0) | 0 (0) |
| *Patiria miniata* | Echinodermata | Echinoderms | 0 | 0.3 (0) | 0 (0) | 0.1 (0.1) | 0 (0) |
| *Pisaster ochraceus* | Echinodermata | Echinoderms | 0.7 | 0 (0) | 0 (0.1) | 0 (0) | 0 (0) |
| *Strongylocentrotus purpuratus* | Echinodermata | Echinoderms | 11.3 | 1.3 (0.5) | 0.6 (0.7) | 0.9 (0.9) | 7.1 (5.6) |
| *Chama pellucida* | Mollusca | Bivalves | 0.4 | 0 (0) | 0 (0) | 0 (0) | 0 (0) |
| *Chlamys hastata* | Mollusca | Bivalves | 0 | 0 (0) | 0 (0) | 0 (0) | 0 (0) |
| *Gari californica* | Mollusca | Bivalves | 0 | 0 (0) | 0 (0) | 0 (0) | 0 (0) |
| *Kellia laperousii* | Mollusca | Bivalves | 0.4 | 0 (0) | 0 (0) | 0 (0) | 0 (0) |
| *Modiolus spp* | Mollusca | Bivalves | 0 | 0 (0) | 0 (0) | 0 (0) | 0 (0) |
| *Mytilus californianus* | Mollusca | Bivalves | 9.3 | 0.3 (0.2) | 0.2 (0.1) | 3.2 (2.7) | 87.2 (88.6) |
| *Pseudochama exogyra* | Mollusca | Bivalves | 0 | 0 (0) | 0 (0) | 0 (0) | 0 (0) |
| *Acanthinucella punctulata* | Mollusca | Gastropods | 0 | 0.5 (0.5) | 0.2 (0.1) | 0.2 (0.2) | 0.8 (0.5) |
| *Acmaea mitra* | Mollusca | Gastropods | 0.6 | 0.1 (0.1) | 0.1 (0.1) | 0.2 (0.1) | 0.2 (0.4) |
| *Alia carinata* | Mollusca | Gastropods | 122.4 | 5 (1.6) | 0.8 (0.5) | 0.6 (0.8) | 0.9 (1.1) |
| *Amphissa columbiana* | Mollusca | Gastropods | 0 | 0 (0.1) | 0 (0) | 0 (0) | 0 (0) |
| *Amphissa versicolor* | Mollusca | Gastropods | 9.1 | 2.2 (1.5) | 0.2 (0.1) | 0.6 (0.5) | 3 (3.5) |
| *Antisabia panamensis* | Mollusca | Gastropods | 0 | 0 (0) | 0 (0) | 0 (0) | 0 (0) |
| *Aplysia californica* | Mollusca | Gastropods | 0 | 0 (0) | 0 (0) | 0 (0) | 0 (0) |
| *Atrimitra idae* | Mollusca | Gastropods | 0 | 0 (0) | 0 (0) | 0 (0) | 0.1 (0.1) |
| *Californiconus californicus* | Mollusca | Gastropods | 0 | 0 (0) | 0 (0) | 0.1 (0.1) | 0.5 (0.4) |
| *Calliostoma annulatum* | Mollusca | Gastropods | 0 | 0.3 (0.4) | 0 (0) | 0 (0) | 0 (0) |
| *Calliostoma canaliculatum* | Mollusca | Gastropods | 0 | 0 (0) | 0 (0) | 0 (0) | 0 (0) |
| *Calliostoma ligatum* | Mollusca | Gastropods | 0.5 | 1.2 (0.5) | 1 (0.6) | 0.9 (0.4) | 0.4 (0.3) |
| *Ceratodoris rosacea* | Mollusca | Gastropods | 0.2 | 0 (0) | 0 (0) | 0 (0) | 0 (0) |
| *Coryphella trilineata* | Mollusca | Gastropods | 0 | 0 (0) | 0 (0) | 0 (0) | 0 (0) |
| *Crepidula adunca* | Mollusca | Gastropods | 11.3 | 19.5 (5.4) | 19.6 (6.9) | 22.1 (10.5) | 20.6 (6.5) |
| *Diaphoreolis lagunae* | Mollusca | Gastropods | 0 | 0 (0) | 0 (0) | 0 (0) | 0 (0) |
| *Epitonium indianorum* | Mollusca | Gastropods | 0 | 0 (0) | 0 (0) | 0 (0) | 0 (0) |
| *Epitonium tinctum* | Mollusca | Gastropods | 0 | 0.2 (0) | 0 (0) | 0 (0.1) | 0.1 (0.1) |
| *Eulithidium spp* | Mollusca | Gastropods | 0 | 0 (0) | 0 (0) | 0 (0) | 0 (0) |
| *Fissurella volcano* | Mollusca | Gastropods | 0.7 | 1.7 (0.5) | 0.4 (0.2) | 0.5 (0.3) | 0.4 (0.4) |
| *Hermissenda spp* | Mollusca | Gastropods | 0 | 0.2 (0.1) | 0 (0) | 0 (0) | 0 (0) |
| *Hesperaptyxis luteopictus* | Mollusca | Gastropods | 0 | 0.1 (0) | 0.1 (0.2) | 0 (0) | 0 (0) |
| *Hespererato vitellina* | Mollusca | Gastropods | 0 | 0.2 (0.1) | 0 (0) | 0 (0) | 0 (0) |
| *Kelletia kelletii* | Mollusca | Gastropods | 0 | 0 (0) | 0 (0) | 0 (0) | 0 (0) |
| *Lacuna marmorata* | Mollusca | Gastropods | 0 | 3.5 (1.6) | 1.5 (1.4) | 0 (0) | 0 (0) |
| *Lacuna porrecta* | Mollusca | Gastropods | 0 | 0 (0) | 0 (0) | 0 (0) | 0 (0) |
| *Lacuna unifasciata* | Mollusca | Gastropods | 0 | 0 (0) | 0 (0) | 0 (0) | 0 (0) |
| *Limacia cockerelli* | Mollusca | Gastropods | 0 | 0 (0) | 0 (0) | 0 (0) | 0 (0) |
| *Littorina keenae* | Mollusca | Gastropods | 0 | 0.1 (0.1) | 0 (0) | 0 (0) | 0 (0) |
| *Littorina scutulata/plena* | Mollusca | Gastropods | 0 | 0.3 (0.3) | 0.1 (0.2) | 0.1 (0.1) | 0.4 (0.2) |
| *Lottia digitalis* | Mollusca | Gastropods | 0 | 0.2 (0.1) | 0 (0) | 0 (0) | 0.1 (0.2) |
| *Lottia instabilis* | Mollusca | Gastropods | 0 | 0 (0) | 0 (0) | 1.1 (1) | 0 (0) |
| *Lottia limatula* | Mollusca | Gastropods | 5.5 | 1.5 (0.7) | 0.3 (0.3) | 3.5 (3.2) | 1 (0.4) |
| *Lottia scabra* | Mollusca | Gastropods | 0.1 | 1.7 (1.6) | 2.9 (6) | 1.4 (2.4) | 0.4 (0.6) |
| *Lottia scutum* | Mollusca | Gastropods | 33.1 | 0.2 (0.2) | 0.2 (0.1) | 0 (0) | 0.1 (0.1) |
| *Margarites salmoneus* | Mollusca | Gastropods | 0 | 0 (0) | 0 (0) | 0 (0) | 0 (0) |
| *Mitrella tuberosa* | Mollusca | Gastropods | 0 | 0 (0) | 0 (0) | 0 (0) | 0 (0) |
| *Nassarius mendicus* | Mollusca | Gastropods | 0 | 0 (0) | 0 (0) | 0 (0) | 0 (0) |
| *Nucella spp* | Mollusca | Gastropods | 0 | 0 (0) | 0 (0) | 0 (0) | 0 (0) |
| *Odetta fetella* | Mollusca | Gastropods | 0 | 0 (0) | 0 (0) | 0 (0) | 0 (0) |
| *Onchidella carpenteri* | Mollusca | Gastropods | 0 | 0 (0) | 0 (0) | 0 (0) | 0 (0) |
| *Paciocinebrina circumtexta* | Mollusca | Gastropods | 0 | 1 (0.2) | 0.5 (0.3) | 0.7 (0.2) | 2.2 (0.6) |
| *Petaloconchus montereyensis* | Mollusca | Gastropods | 0 | 0 (0) | 0 (0) | 0 (0) | 0 (0) |
| *Pseudopusula californiana* | Mollusca | Gastropods | 0 | 0 (0) | 0 (0) | 0 (0) | 0 (0) |
| *Rostanga pulchra* | Mollusca | Gastropods | 0.2 | 0 (0.1) | 0 (0) | 0 (0) | 0 (0) |
| *Tectura paleacea* | Mollusca | Gastropods | 0 | 0.7 (0.1) | 0.1 (0.1) | 0 (0) | 0 (0) |
| *Tegula brunnea* | Mollusca | Gastropods | 0.1 | 30.6 (6) | 7.5 (4.3) | 9.1 (3) | 8 (5.4) |
| *Tegula funebralis* | Mollusca | Gastropods | 47.1 | 150.5 (8.2) | 124.5 (40.4) | 271.7 (32.7) | 286.5 (29.3) |
| *Tegula montereyi* | Mollusca | Gastropods | 0 | 0 (0) | 0 (0) | 0 (0) | 0 (0) |
| *Tegula pulligo* | Mollusca | Gastropods | 0 | 0.5 (0.7) | 0 (0) | 0 (0) | 0.1 (0.1) |
| *Thylacodes squamigerus* | Mollusca | Gastropods | 0 | 10.9 (5) | 7.5 (5.1) | 1.6 (1) | 1.2 (0.9) |
| *Urosalpinx cinerea* | Mollusca | Gastropods | 0 | 0 (0) | 0 (0) | 0 (0) | 0 (0) |
| *Cyanoplax hartwegii* | Mollusca | Polyplacophorans | 0.1 | 0.1 (0) | 0 (0) | 0 (0) | 0 (0) |
| *Lepidozona spp* | Mollusca | Polyplacophorans | 0 | 0 (0) | 0 (0) | 0 (0) | 0 (0) |
| *Mopalia muscosa* | Mollusca | Polyplacophorans | 0.6 | 0.3 (0.1) | 0 (0) | 0.1 (0.1) | 0.4 (0.3) |
| *Nuttallina californica* | Mollusca | Polyplacophorans | 0 | 0.1 (0.2) | 0.2 (0.2) | 0.1 (0.2) | 0.1 (0.1) |
| *Tonicella lineata* | Mollusca | Polyplacophorans | 0 | 0.1 (0) | 0 (0) | 0.1 (0.1) | 0.1 (0.1) |
| *Emplectonema gracile* | Nemertea | Nemerteans | 0 | 0.4 (0.6) | 0 (0) | 0 (0) | 0 (0) |
| *Notocomplana acticola* | Platyhelminthes | Platyhelminths | 0 | 0 (0) | 0 (0) | 0 (0) | 0.2 (0.3) |
| *Haliclona spA* | Porifera | Poriferans | 0 | 0 (0) | 0 (0) | 0 (0) | 0.5 (0.4) |
