# Supplementary material for: Persistent loss of animal diversity on a rocky shore over nine decades and across multiple investigators
Source: PeerJ. 2026 Apr 16;14:e21099. doi: 10.7717/peerj.21099 (PMC13092228; doi:10.7717/peerj.21099)
Supplement: Supplemental Information 3 [file peerj-14-21099-s003.docx]

**Table S3: List of processing steps and associated observations (i.e., total abundance) to prepare the quantitative dataset for analysis.**

| **Processing step** | **Observations** | **Proportion** |
| --- | --- | --- |
| All observations | 347465 | 1 |
| Removed taxa treated as qualitative by Hewatt | 329009 | 0.947 |
| Removed limpets on *Tegula* | 328266 | 0.945 |
| Removed taxa above genus | 328110 | 0.944 |
| Removed genera represented by multiple species | 327415 | 0.942 |
