# Supplementary figures and images for: Persistent loss of animal diversity on a rocky shore over nine decades and across multiple investigators
Source: PeerJ. 2026 Apr 16;14:e21099. doi: 10.7717/peerj.21099 (PMC13092228; doi:10.7717/peerj.21099)

Vertical relief (m)

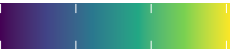

0.2 0.4 0.6 0.8

Quadrat 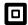 core 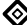 extra

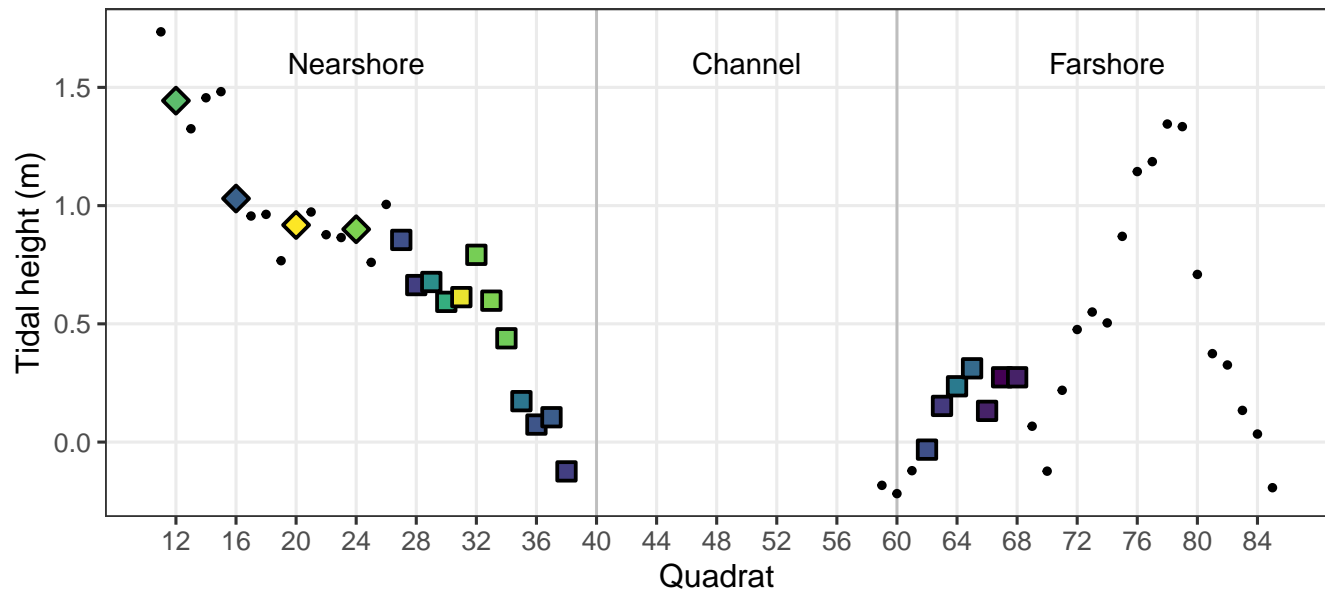

Supplement: Supplemental Information 4 — The historical transect begins at the uppermost limits of the rocky shore and is set perpendicular to the shoreline. Nearshore quadrats are separated from farshore quadrats by a channel at minus tides. Square symbols represent ’core’ quadrats that were resurveyed from 1993 to 2023 and diamonds represent ’extra’ quadrats that were resurveyed from 2020 to 2023; small dots represent quadrats that have not been resurveyed but for which we have tidal height and vertical relief data. Vertical relief was measured as the maximum vertical distance within a quadrat. The historical data archived by (Hewatt, 1937) included quadrats 11-100, 105. [file peerj-14-21099-s004.pdf]

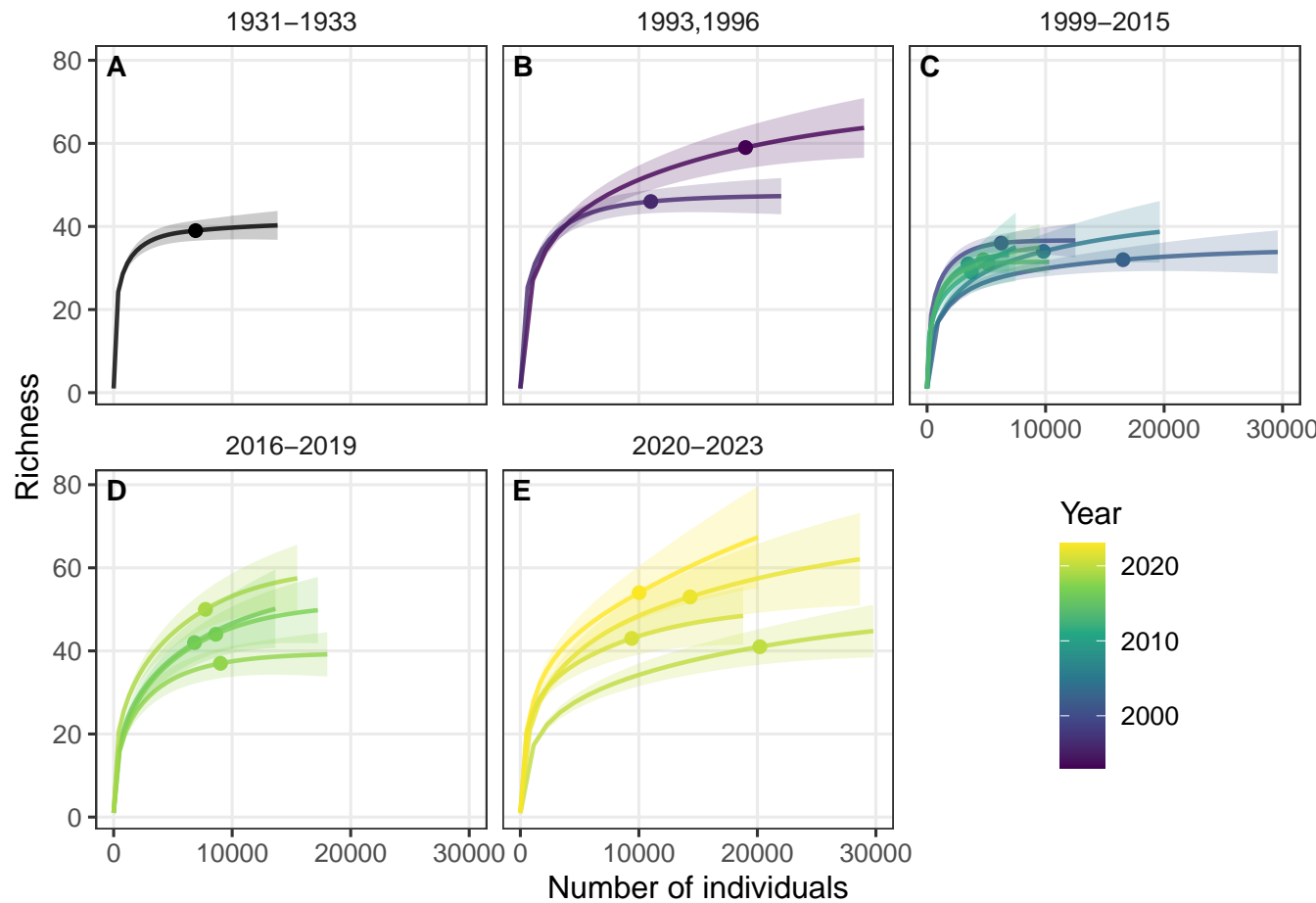

Supplement: Supplemental Information 5 — The observed richness is plotted as a point; lines before the point represent rarefaction and lines beyond the point represent extrapolation. Shaded areas represent 95% confidence intervals. [file peerj-14-21099-s005.pdf]

● 1931–1933    ● 1993,1996    ● 1999–2015    ● 2016–2019    ● 2020–2023

mobile

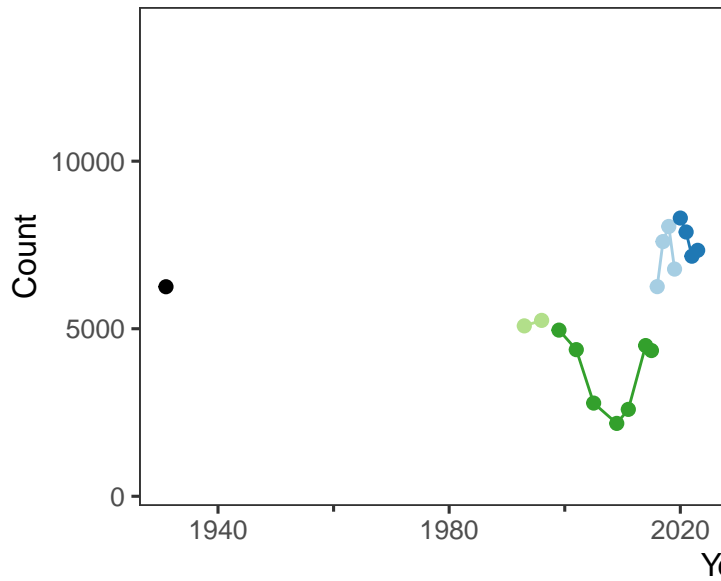

sessile

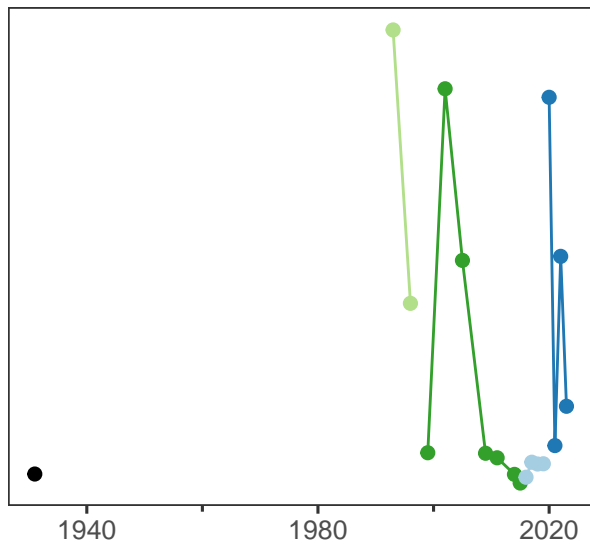

Supplement: Supplemental Information 6 [file peerj-14-21099-s006.pdf]

● 1931–1933 ● 1993,1996 ● 1999–2015 ● 2016–2019 ● 2020–2023

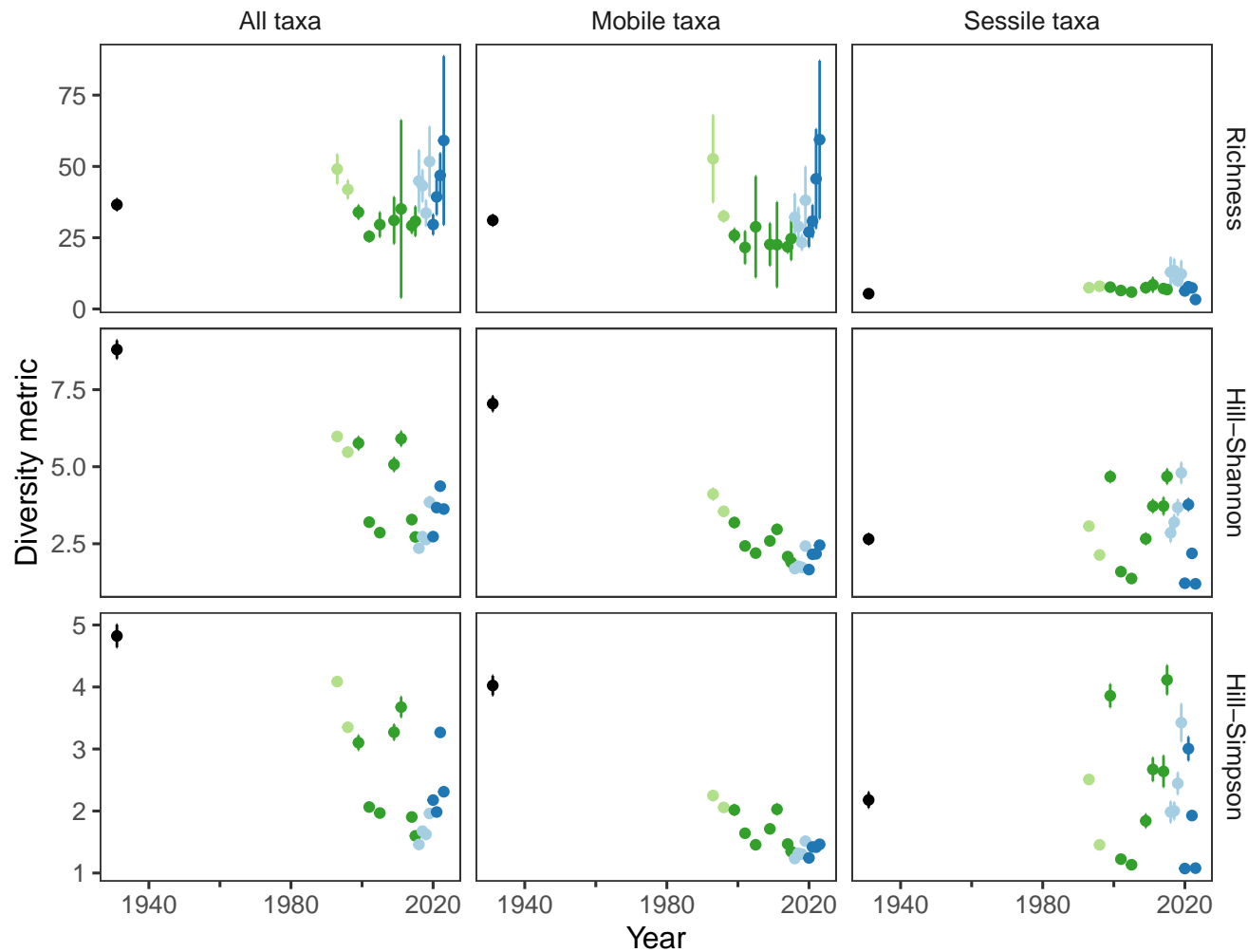

Supplement: Supplemental Information 7 — Diversity metrics were estimated using a coverage-based estimator (estimate ± 95% confidence intervals). Note that panels for all taxa are duplicated from Fig. 3A-C from the main text. [file peerj-14-21099-s007.pdf]
