# Supplementary material for: Persistent loss of animal diversity on a rocky shore over nine decades and across multiple investigators
Source: PeerJ. 2026 Apr 16;14:e21099. doi: 10.7717/peerj.21099 (PMC13092228; doi:10.7717/peerj.21099)
Supplement: Supplemental Information 9 [file peerj-14-21099-s009.pdf]

Elahi, R., F. Micheli, J. Watanabe. 2026. Persistent loss of animal diversity on a rocky shore over nine decades and across multiple investigators. PeerJ.

Appendix S2: Statistical models

Model 1: Estimating linear trends in biodiversity

We used the following statistical model for inference and estimation of linear trends in biodiversity responses in the context of investigator effects. We modeled richness (b_i) in each year (centered on 2008; y_i) and investigator era (Era[j], for $j = 1..4$) as:

$$
\begin{aligned}
b_i &\sim \text{Normal}(\mu_i, \sigma) \\
\mu_i &= \alpha_{\text{Era}[i]} + \beta y_i \\
\boldsymbol{\alpha}_j &\sim \text{Normal}(50, 25), \quad \text{for } j = 1..4 \\
\beta &\sim \text{Normal}(0, 1) \\
\sigma &\sim \text{Exponential}(1)
\end{aligned}
$$

We used the same model for two diversity metrics and species evenness, but with different weakly informative prior probability distributions. For Hill-Shannon diversity, $\boldsymbol{\alpha}_j \sim \text{Normal}(6, 3)$ and $\beta \sim \text{Normal}(0, 0.2)$. For Hill-Simpson diversity, $\boldsymbol{\alpha}_j \sim \text{Normal}(4, 2)$ and $\beta \sim \text{Normal}(0, 0.2)$. For evenness, $\boldsymbol{\alpha}_j \sim \text{Normal}(0.5, 0.3)$ and $\beta \sim \text{Normal}(0, 0.02)$. Prior predictive plots were used to ensure that a wide range of linear outcomes were plausible.

Model 2: Estimating the causal effect of seawater temperature on Hill-Shannon diversity

We used the following statistical model to estimate the causal effect of maximum sea surface temperature on Hill-Shannon diversity. We modeled Hill-Shannon diversity (D_i) as a function of the previous year's maximum sea surface temperature (standardized; c_i), year (centered on 2008; y_i) and investigator era (Era[j], for $j = 1..4$) as:

$$
\begin{aligned}
D_i &\sim \text{Normal}(\mu_i, \sigma) \\
\mu_i &= \alpha_{\text{Era}[i]} + \beta_c c_i + \beta_y y_i \\
\boldsymbol{\alpha}_j &\sim \text{Normal}(6, 3), \quad \text{for } j = 1..4 \\
\beta_c &\sim \text{Normal}(0, 0.5) \\
\beta_y &\sim \text{Normal}(0, 0.5) \\
\sigma &\sim \text{Exponential}(1)
\end{aligned}
$$

Model 3: Estimating the causal effect of seawater temperature on Hill-Shannon diversity: Gaussian process

We also used a Gaussian process model to estimate the causal effect of maximum sea surface temperature on Hill-Shannon diversity. We modeled Hill-Shannon diversity (D_i) as a function of the previous year's maximum sea surface temperature (standardized; c_i) and temporal autocorrelation between years. We expected that closer years would share unmeasured influences on diversity and thus we estimated the covariance matrix (K_{ij}, for $j = 1..17$) between all pairs of the survey years from 1993 to 2023 (n = 17). We assumed that the covariance between any two years i and j declined exponentially with the squared number of years between them. The equation for the covariance matrix (K_{ij}) has two parameters: ρ determines the rate of exponential decline; η is the maximum covariance between any two years. The full model description is:

$$
\begin{aligned}
D_i &\sim \text{Normal}(\mu_i, \sigma) \\
\mu_i &= \bar{\alpha} + \alpha_{\text{Year}[i]} + \beta c_i \\
\bar{\alpha} &\sim \text{Normal}(6, 3) \\
\boldsymbol{\alpha}_{1:j} &\sim \text{MVNormal}(\mathbf{0}, \mathrm{K}) \\
\mathrm{K}_{ij} &= \eta^2 \exp(-\rho^2 \mathrm{Y}_{ij}^2) \\
\eta^2 &\sim \text{Exponential}(1) \\
\rho^2 &\sim \text{Exponential}(2) \\
\beta &\sim \text{Normal}(0, 0.5) \\
\sigma &\sim \text{Exponential}(1)
\end{aligned}
$$

We used a non-centered parameterization of the above model to improve Markov chain sampling. See section 14.5 in McElreath (2020) for an analogous example of spatial autocor-

relation.

Model 4: Estimating the effect of geographic range on population change

We used the following statistical model to estimate the effect of geographic range (Coastwide, Southern, Uncertain; $\boldsymbol{\alpha}_j$) on population change (log response ratio). The mean log response ratio ($R_{\text{obs},i}$) for each taxon (i) was associated with a standard error ($R_{\text{SE},i}$). We incorporated this measurement error by modeling the unobserved true log response ratios for each taxon ($R_{\text{true},i}$):

$$
\begin{aligned}
R_{\text{obs},i} &\sim \text{Normal}(R_{\text{true},i}, R_{\text{SE},i}) \\
R_{\text{true},i} &\sim \text{Normal}(\mu_i, \sigma) \\
\mu_i &= \alpha_{\text{Range}[i]} \\
\boldsymbol{\alpha}_j &\sim \text{Normal}(0, 2), \quad \text{for } j = 1..3 \\
\sigma &\sim \text{Exponential}(1)
\end{aligned}
$$

References

McElreath, Richard. 2020. Statistical Rethinking: A Bayesian Course with Examples in R and Stan. CRC Press.
